# Immunogenicity of poxvirus A16/G9 entry–fusion subcomplex and its restriction by A56/K2 protein informs vaccine design

**DOI:** 10.1038/s41564-026-02392-6

**Published:** 2026-06-10

**Authors:** Huibin Yu, Wolfgang Resch, Catherine A. Cotter, Wei Xiao, Tase Karamanolis, Ahmed A. Belghith, Maxinne A. Ignacio, Patricia L. Earl, Gary H. Cohen, Bernard Moss

**Affiliations:** 1 Laboratory of Viral Diseases, NIAID, NIH, Bethesda, MD USA; 2 Center for Information Technology, NIH, Bethesda, MD USA; 3 Department of Microbiology, School of Dental Medicine, University of Pennsylvania, Philadelphia, PA USA

**Keywords:** Viral immune evasion, Experimental models of disease

## Abstract

Poxviruses rely on a conserved multiprotein entry–fusion complex, providing numerous potential antibody targets, although the immunogenicities of only a few have been analysed. Here we tested the ectodomains of ten orthopoxvirus entry–fusion complex proteins in rabbit and mouse immunization experiments and determined that six induce neutralizing antibodies. Focusing on the apical A16/G9 heterodimer, we show that it induces antibodies that prevent vaccinia virus (VACV) entry, cross-neutralize cowpox virus (CPXV) and monkeypox virus (MPXV), and protect mice against lethal challenge infections with VACV and CPXV. However, antibodies to A16/G9 had limited detection following infections with attenuated or virulent VACV, CPXV or MPXV, but were robustly induced upon immunization with recombinant VACVs secreting G9 or that have deletions of the genes encoding the viral fusion suppressor A56/K2 complex, which specifically binds A16/G9. Our work identifies previously unrecognized immunogens for incorporation into recombinant vaccines and characterizes a mode of immune evasion via the A56/K2 complex.

## Abstract (web summary)

The poxvirus A16/G9 entry protein is shown to be a potent cross-protective antigen for incorporation into recombinant vaccines, but its immunogenicity in current live vaccines is restricted by the viral A56/K2 fusion suppressor complex.

## Main

The 2022 global resurgence of human mpox, caused by monkeypox virus (MPXV), and the increased incidence of mpox in Africa have spurred efforts to develop improved vaccines because the current vaccine induces low and non-durable neutralizing antibodies against MPXV^1,2^ and incomplete protection against infection, although severe disease is reduced^3^. The development of alternative recombinant vaccines requires intimate knowledge of poxvirus biology and the identification of immunologically optimal targets^4,5^.

The entry proteins of enveloped viruses are commonly the major targets of protective antibodies. Unlike other viruses, poxviruses have membrane envelopes that are assembled within the cytoplasm so that the entry–fusion proteins are not displayed on the plasma membrane where they could induce antibodies. Furthermore, whereas other viruses utilize one or a few viral proteins to mediate fusion, poxviruses express 11 highly conserved proteins (Extended Data Table 1) that are each essential and form the entry–fusion complex (EFC)^6,7^. The structures of the individual EFC ectodomains (except the smallest component) and three dimeric subcomplexes have been experimentally determined^5^. Comparisons of the EFC protein structures with all proteins in the Protein Data Bank (PDB) and the database of AlphaFold models reveal no similarities, suggesting that these EFC proteins adopt unique folds^8^. Nine of the EFC proteins are required for assembly of the complex forming the core structure^9^, which has been modelled using AlphaFold2 (ref. ^5^) and resolved by cryo-electron microscopy^10^. Although the definitive fusion peptide(s) have not been identified, the A16/G9 proteins at the apex of the EFC are specifically bound by viral fusion suppressor proteins A26 (ref. ^11^) and the A56/K2 heterodimer^12^.

Each EFC protein is a potential immune target, but only two peripheral proteins and one core protein^5^ were previously reported to induce neutralizing antibodies. The present study aimed to identify additional targets of neutralizing antibodies that have not been found by analysing immune serum with protein microarrays^13^ or by screening large monoclonal antibody libraries^14^. We identified previously unrecognized immunogens that could induce cross-protective neutralizing antibodies to orthopoxviruses (OPXVs), a key step towards developing more effective vaccines. We found that virus-neutralizing antibodies to the conserved A16/G9 heterodimer of the EFC were robustly induced by the purified recombinant protein but seldom by infection or vaccination. The ability of antibodies to neutralize virus but not be induced by virus infection illustrates a distinction between antigenicity and immunogenicity. Evasion of immunogenicity is related to concealment of the complex within the viral membrane, as demonstrated by engineering recombinant VACV to induce antibodies either by secreting a soluble form of the protein or by preventing expression of the viral A56/K2 fusion suppressor leading to the insertion and exposure of the EFC including A16/G9 in the cell membrane. These insights into the biology of OPXVs may advance next generation vaccine design. A preliminary version of this study was published as a preprint^15^.

## Results

### Neutralizing activities of antisera correlate with binding to intact virions

**Fig. 1:**
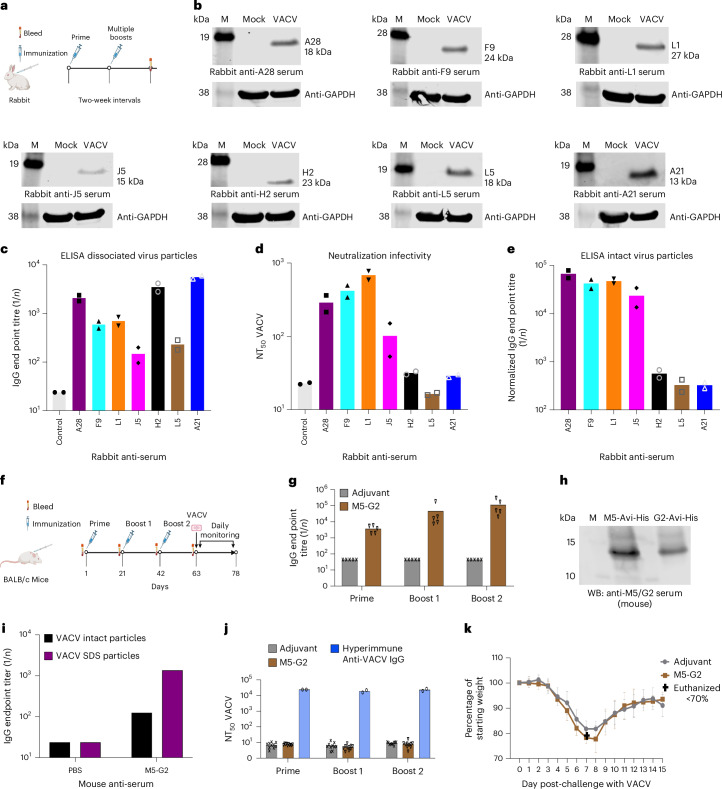
Binding and neutralizing activities of antibodies to EFC proteins. **a**, Serum from male white New Zealand rabbits that were immunized with individual purified EFC proteins (Extended Data Fig. 1) at 2-week intervals were used for immunoassays. **b**, SDS–PAGE blots of lysates of mock- or VACV-infected cells were probed with individual antisera. EFC protein masses are shown on the right. M refers to marker proteins, with masses on the left. GAPDH was analysed as a loading control. **c**–**e**, Antiserum from two rabbits was analysed by measuring binding to purified VACV dissociated with SDS, renatured and adsorbed onto ELISA plate wells (**c**); by assessing neutralization of VACV infectivity (**d**); and by ELISA against intact VACV particles after normalization to the titre for dissociated virions (**e**). **f**, The experimental plan for immunization of female BALB/c mice (total *n* = 10) with M5/G2 and challenge with 10^5^ p.f.u. of VACV. **g**, ELISA after priming and boosting of five mice. **h**, SDS–PAGE blots of purified M5-Avi-His and G2-Avi-His probed with anti-M5/G2 serum. **i**, Binding of pooled (*n* = 10) anti-M5/G2 mouse serum to intact and SDS-disrupted VACV particles. **j**, The absence of VACV-neutralizing antibody in serum from individual M5/G2 immunized mice (*n* = 10). IgG from VACV immunized rabbit was determined in duplicate as a positive control. **k**, Weight loss of individual female mice (*n* = 10) following challenge with VACV. Geometric mean values ± s.d. are shown. Experiments in **b**–**e** and **g**–**i** were repeated with similar results. Schematic in **a** and **f** created in BioRender; Yu, H. https://biorender.com/fmctsd0 (2026). Source data

**Extended Data Fig. 1:**
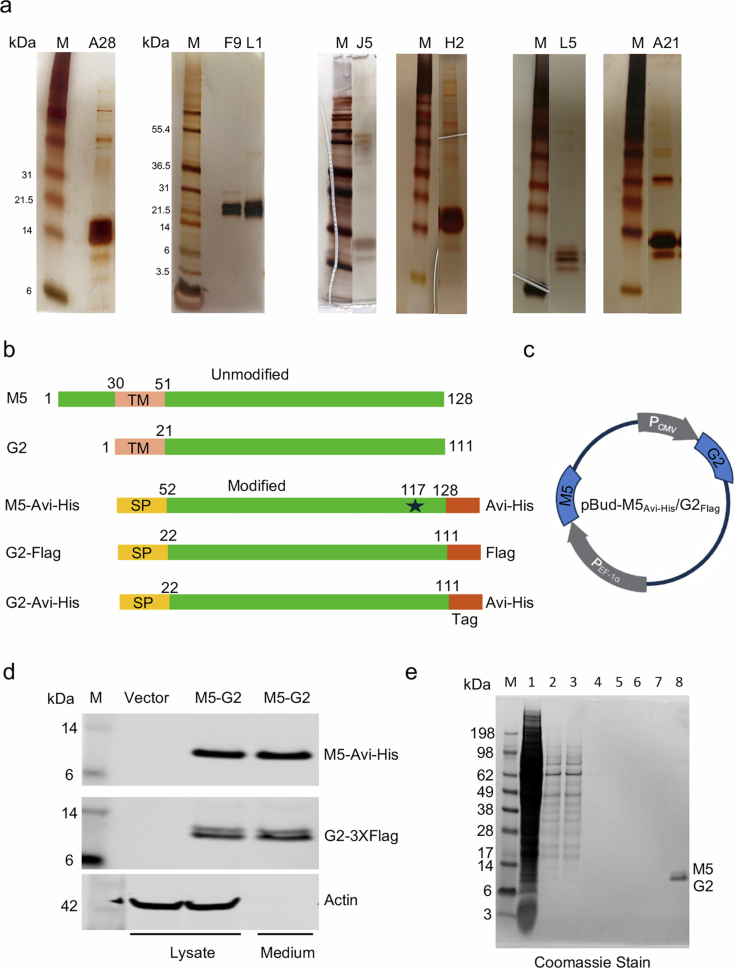
Analysis of purified EFC proteins. **a**. Secreted A28, F9, L1, J5, H2, L5 and A21 EFC proteins were expressed by baculoviruses, concentrated from clarified serum-free medium, purified with Ni-NTA beads, analyzed by SDS-PAGE and visualized by silver staining. M, marker proteins with masses in kDa. Representative data from one of two rabbits. **b**. Mammalian codon-optimized DNA constructs encoding M5 residues 52-128 (UniProt accession number PG099_MONPV) and G2 residues 22-111 (UniProt accession number A0A0F6N9M0_MONPV) with an N-terminal human modified albumin signal peptide^46^ and C-terminal epitope tags were chemically synthesized. Asterisk indicates mutation of a potential N-glycosylation site. **c**. pBudCE4.1 dual promoter plasmid (Thermo Fisher Scientific) for expression of M5 and G2. **d**. The expression plasmid was transfected into Expi293F cells and the lysate and medium were analyzed by immunoblotting with anti-His and anti-Flag antibodies. Anti-actin antibody was used as a loading control. Representative of replicate experiments shown. **e**. Concentrated medium was loaded on Ni-NTA beads, and the unbound and imidazole eluted fractions were analyzed by SDS-PAGE and Coomassie Blue staining. Lanes: (1) cell lysate, (2) medium, (3) flow through and (4-6) wash, (7, 8) eluate. M5 and G2 co-migrate as they are of similar molecular mass. M, marker proteins with masses in kDa. Representative of replicate experiments shown.

To systematically assess the immunogenicity of individual EFC proteins, we first expressed the secreted ectodomains of seven proteins in insect Sf9 cells as described previously^16^. Rabbits were immunized with the individual purified proteins (Fig. 1a and Extended Data Fig. 1a) and specific antibodies in the sera were identified by western blotting of VACV-infected cell lysates (Fig. 1b) and by binding to proteins of dissociated purified virions (Fig. 1c). Anti-A28, anti-F9, anti-L1 and anti-J5 sera neutralized VACV, but neutralization activity remained near baseline for the anti-H2, anti-L5 and anti-A21 sera (Fig. 1d), despite good antibody binding to dissociated virus particles. We also evaluated the binding of the antisera to intact virions. The same proteins that induced neutralizing antibodies also exhibited the highest binding to intact virions (Fig. 1e).

VACV L5 and the G3 EFC protein form a stable dimer^17^, which might enhance immunogenicity. Nevertheless, the purified heterodimer of the highly conserved MPXV homologues M5 and G2 (Extended Data Table 1 and Extended Data Fig. 1b–e) induced antibodies in mice that bound well to the antigen and to dissociated virus particles (Fig. 1f–h) but poorly to intact virus (Fig. 1i) and failed to neutralize virus or protect mice against weight loss following challenge with VACV (Fig. 1j,k). Thus, of the eight EFC proteins analysed so far, only A28, F9, L1 and J5 induced neutralizing antibodies.

### A16/G9 heterodimer induces potent neutralizing antibodies

**Extended Data Fig. 2:**
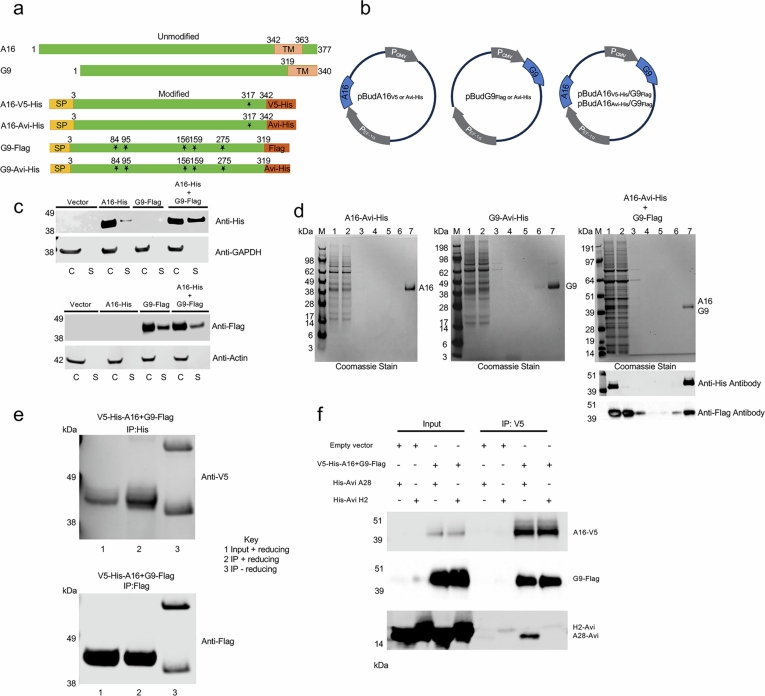
Expression, purification and interactions of A16 and G9 proteins. **a**. Mammalian codon-optimized DNA constructs encoding residues 3-342 of A16 (UniProt accession number P16710 · A16_VACCW) and residues 3-319 of G9 (UniProt accession number P07611 · G9_VACCW) with an N-terminal human modified albumin signal peptide and C-terminal epitope tags were chemically synthesized. A potential N-glycosylation site at threonine 317 of A16 was mutated to valine and serines and threonines at residues 84, 95, 156, 159, and 275 of G9 were mutated to alanine. **b**. Modified ORFs were inserted into the pBudCE4.1 dual expression vector. **c**. Immunoblots of cell lysates (C) and secreted proteins (S) following transfection of plasmids into 293 T cells. Primary antibodies used to probe blots are indicated on the right. **d**. Purification of proteins on Ni-NTA beads. SDS-PAGE of starting material (1), flow through (2), wash (3-5) and elution (6, 7) fractions detected by Coomassie blue staining and with anti-His and anti-Flag antibodies. Marker proteins (M). **e**. Detection of secreted A16/G9 dimer from 293 T cells transfected with plasmid expressing A16-V5-His+G9-3XFlag after SDS treatment in the presence or absence of a reducing agent. **f**. Co-immunoprecipitation of His-Avi A28, but not H2, with V5-His-A16 + G9-Flag. Western blot shown. For c, d, e representative of replicate experiments shown.

Next, we tested the immunogenicity of A16 and G9, which form a stable heterodimer^12^. Expression plasmids were constructed encoding the modified proteins with transmembrane domains deleted, signal peptides and epitope tags added, and predicted N-glycosylation sites mutated (Extended Data Fig. 2a,b). While A16 remained mostly cell associated when expressed alone in Expi293F cells, its secretion increased when co-expressed with G9, suggesting an intracellular interaction between the two proteins (Extended Data Fig. 2c). By contrast, G9 was secreted to a similar extent either when expressed alone or together with A16. Thus, G9 acts as a chaperone that assists the folding and secretion of A16. Co-expressed A16 and G9 formed a heterodimer as determined by co-purification and co-electrophoresis under non-reducing conditions (Extended Data Fig. 2d,e).

**Extended Data Fig. 3:**
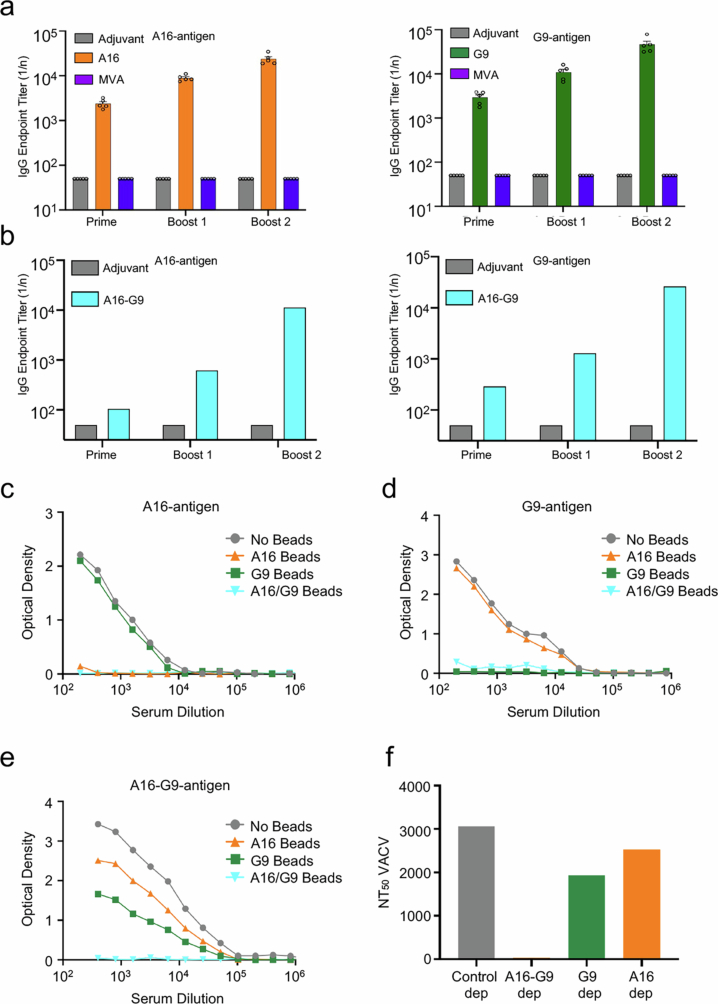
Immunogenicity of A16, G9 and A16/G9 heterodimer. **a**. Groups of female BALB/c mice (n = 5 per group) were primed and boosted at 3-week intervals subcutaneously (SC) with 10 µg of purified A16 or G9 with AddaVax or 10^7^ PFU of MVA intramuscularly (IM) and IgG endpoint binding titre to A16 and G9 antigens determined by ELISA. Bars indicate mean values. **b**. Mice were immunized with A16/G9 as in panel a, and pooled sera from each group were tested by ELISA for binding to A16 and G9 antigens. **c-e**. Depletion of antibodies to A16, G9 and A16/G9. TALON Magnetic Dynabeads (Invitrogen) 4 mg in 100 µl were incubated with 100 µg of purified histidine-tagged A16, G9, or A16/G9 proteins for 4 h at 4 °C. The beads were washed and subsequently incubated overnight with 500 µl of 1:200 diluted A16/G9 serum in PBS at 4 °C. Following incubation, the beads were removed from the serum magnetically. The antibody-depleted sera were then utilized for antibody binding and neutralization assays. **f**. Depletion of VACV neutralizing activity in serum from mice immunized with A16/G9 heterodimers. Panels a,b and f are representative of replicate experiments.

Antibodies in the sera of mice immunized with A16 and G9 bound to the corresponding antigen and increased after successive boosts (Extended Data Fig. 3a). By contrast, no A16- or G9-binding antibodies were detected in sera from mice immunized with modified vaccinia virus Ankara (MVA), a highly attenuated strain used in the currently licensed smallpox/mpox vaccine, suggesting that A16 and G9 have low immunogenicity upon virus infection, as will be confirmed and extended in a later section. Additional experiments demonstrated that antibodies in anti-A16/G9 serum bound to both A16 and G9 (Extended Data Fig. 3b) and that complete depletion of binding was only achieved with the A16/G9 antigen (Extended Data Fig. 3c–e).

**Fig. 2:**
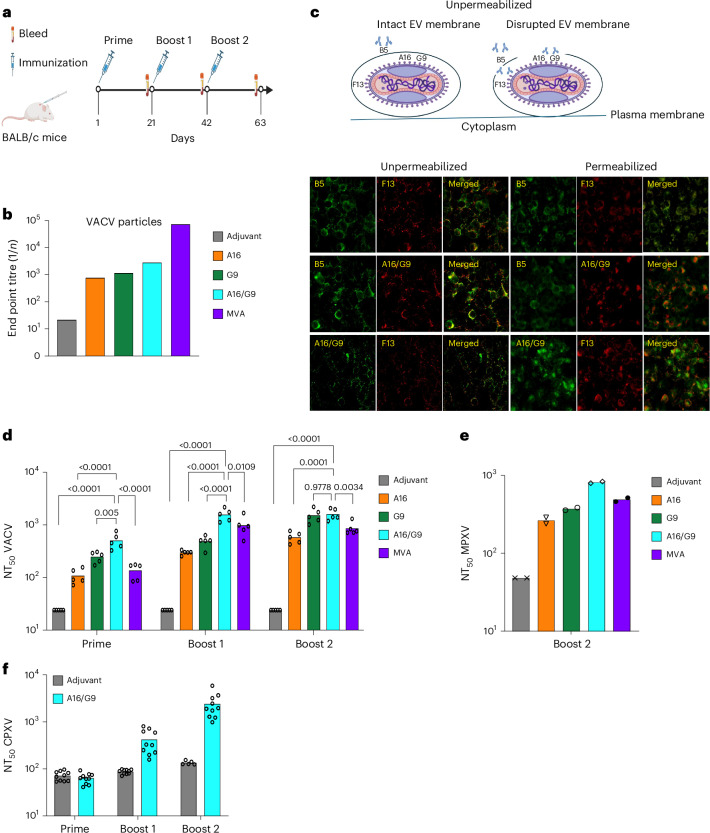
Virus neutralizing and binding activities of A16/G9 antibodies. **a**, Groups of female BALB/c mice (*n* = 5) received three 10-μg doses of A16, G9 or A16/G9 heterodimer subcutaneously with AddaVax or 10^7^ p.f.u. of MVA intramuscularly. **b**, ELISA assessing binding of pooled immune sera to purified virus particles. **c**, Binding of antibodies to progeny virus particles on the surface of infected cells. Top: a diagram of intact EV and disrupted EV showing locations of antigens and accessibility to specific antibodies. Bottom: immunofluorescence staining of unpermeabilized and permeabilized cells with antibodies to EV proteins B5 and F13 and to MV protein A16/G9. **d**, VACV neutralization titre determined with serum of individual mice (*n* = 5) after priming and boosting. Significance to A16/G9 calculated by one-way ANOVA with Dunnett’s multiple comparisons. **e**, MPXV neutralization determined in duplicate with pooled (*n* = 10) serum after boosting. **f**, CPXV neutralization determined with serum of individual mice (*n* = 10) after priming and boosting. Experiments in **b**–**d** were repeated with similar results. Schematic in **a** and **c** created in BioRender; Yu, H. https://biorender.com/fmctsd0 (2026). Source data

Anti-A16/G9 antibodies were able to bind to intact virus particles purified by sucrose gradient sedimentation (Fig. 2b) as well as to virions on the surface of cells (Fig. 2c). Although progeny virus particles on the cell surface are predominantly extracellular enveloped virions (EVs), many have partially disrupted outer membranes, allowing staining of the mature virions (MVs) within^18^ as depicted in Fig. 2c. Antibodies to B5, F13 and A16/G9 bound diffusely within the cytoplasm of permeabilized cells (Fig. 2c), whereas punctate staining of virus particles occurred on the surface of unpermeabilized cells. Anti-B5 antibodies stained all EVs whereas anti-F13 and anti-A16/G9 antibodies stained only partially disrupted ones.

The VACV-neutralizing activity of serum from mice primed and boosted with A16, G9, A16/G9 or MVA was highest in sera from mice immunized with A16/G9, although the difference between G9 and A16/G9 diminished after the second boost (Fig. 2d). A16/G9 induced neutralizing antibodies against both subunits, as only A16/G9 completely depleted neutralizing activity (Extended Data Fig. 3f). The VACV A16, G9 and A16/G9 antisera cross-neutralized MPXV (Fig. 2e) and cowpox virus (CPXV) (Fig. 2f), consistent with the high sequence conservation of EFC proteins.

**Extended Data Fig. 4:**
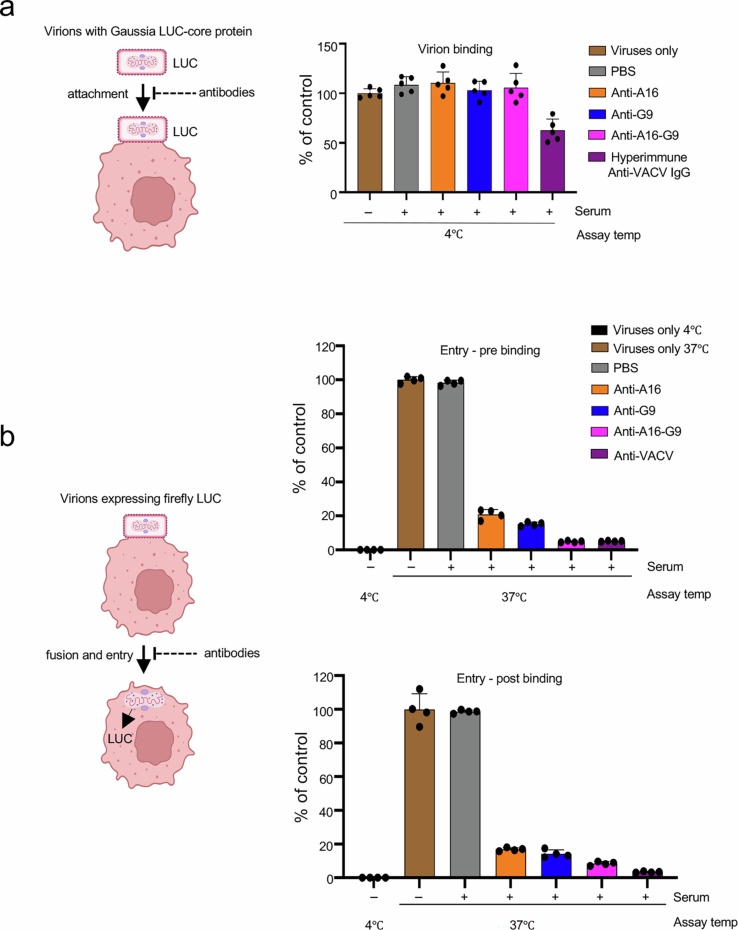
Inhibition of cell entry by anti-A16, -G9 and -A16/G9 antibodies. **a**. Effects of antibodies on binding of VACV MVs to cells. VACV Gauss-A4 virions^41^ (5 PFU/cell) were pre-treated with mouse antisera, or rabbit anti-VACV polyclonal IgG and incubated with HeLa S3 cells in a 96-well plate at neutral pH for 1 h at 4 °C and luciferase activity was measured. Mean values ± SEM of 5 replicates are shown. **b**. Inhibition of virus entry by antibodies to A16/G9. For the pre-attachment inhibition assay diluted serum was incubated with purified WRvFire^20^ at room temperature (RT) prior to virus binding cells and measuring luciferase after 2 h at 37 °C. For the post-attachment inhibition assay HeLa S3 cells were incubated with purified WRvFire (3 PFU/cell) at 4 °C for 30 min to allow attachment and then incubated with dilutions of antibody followed by incubation at 37 °C for 2 h and determination of luciferase. Schematic in **a** and **b** created in BioRender; Yu, H. https://biorender.com/fmctsd0 (2026).

Further studies showed that antibodies to A16, G9 and A16/G9 inhibited virus entry either pre- or post-attachment rather than cell binding (Extended Data Fig. 4a,b).

### Locations of targets of neutralizing and non-neutralizing antibodies within an EFC model

**Fig. 3:**
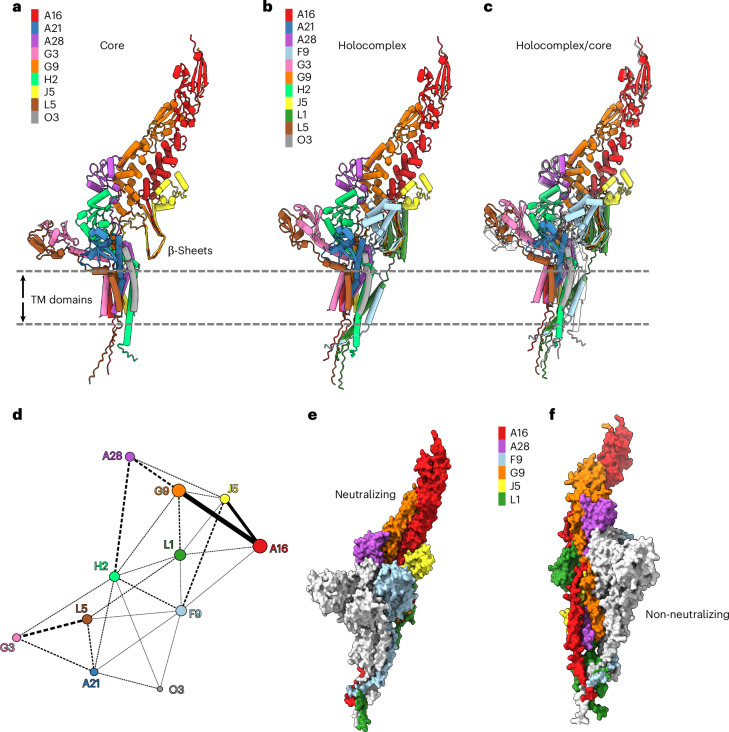
Antibody targets on an EFC model predicted by AlphaFold3. **a**–**c**, Ribbon structural models of the VACV EFC 9-protein core (**a**), 11-protein holocomplex (**b**) and holocomplex superimposed on an uncoloured core (**c**) predicted by AlphaFold3. Individual EFC proteins are identified in the colour keys. **d**, A diagram showing the buried solvent-accessible areas between pairs of proteins. The line widths were manually scaled to the size of the interface. Dotted lines are less than half of the maximum value. **e**, Space-filling model of the EFC holocomplex with neutralizing targets in colour and non-neutralizing targets in grey. Individual neutralizing targets identified in the colour keys. **f**, Same as in **e** except the model is rotated to show an additional perspective. Source data

**Extended Data Fig. 5:**
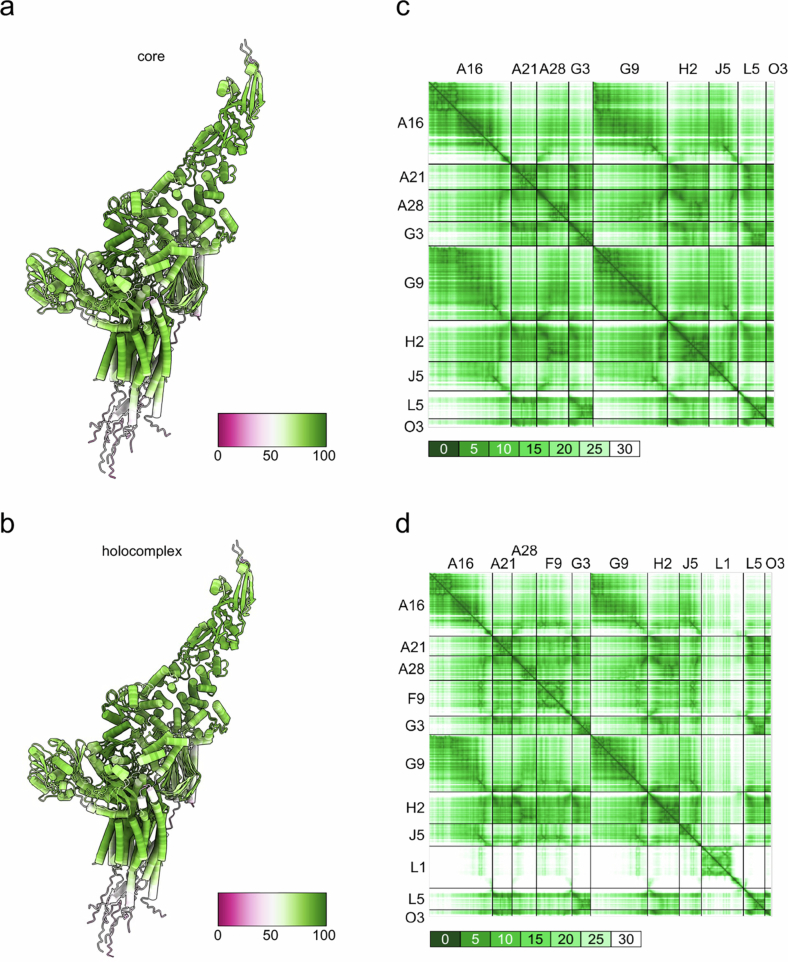
AlphaFold3 model construction and confidence metrics. Models were constructed with AlphaFold version 3.0 using default settings with 5 random seeds per model. The highest ranking model was used for visualizations. The following protein sequences from the VACV WR strain (NC_006998) were used in the AlphaFold predictions. A16:YP_233018.1, A21: YP_233022.1, A28: YP_233033.1, F9: YP_232930.1, G3:YP_232961.1, G9: YP_232969.1, H2: YP_232982.1, J5: YP_232979.1, L1: YP_232970.1, L5: YP_232974.1. Protein structures were visualized using Chimera X 1.9 https://pubmed.ncbi.nlm.nih.gov/37774136/. Superposition of the AlphaFold3 model with experimentally determined structures from PDB (A16:8GP6/A, A21:8U0R/A, A28:8GQO.1/A:30-109, F9:6CJ6/A, G3:7YTU/A, G9:8GP6/B, H2:8INI/A, J5:8WT5.1/A, L1:1YPY/A, L5:7YTU/B) was accomplished using Chimera X’s internal matchmaker command with default settings which carries out an iterative algorithm aligning Cα atoms of matching residues and removing pairs with deviations larger than 2.0 Å resulting in a pruned alignment. Chimera X’s interfaces command was used to calculate the pairwise buried solvent-accessible areas (SASA) of all subunits in the complex. Default settings were used which resulted in a minimum interface size of 300 Å squared when measured with a 1.4 Å probe. **Confidence metrics of predicted core and holocomplex EFC models. a, b**. The predicted local distance test (pLDDT) per-residue measure of local confidence of core and holocomplex models scaled from 0 to 100 with higher score indicating higher confidence. **c, d**. Predicted aligned error (PAE) measure of confidence in relative position of residues within predicted core and holocomplex. models. Regions of high confidence are dark green.

**Extended Data Fig. 6:**
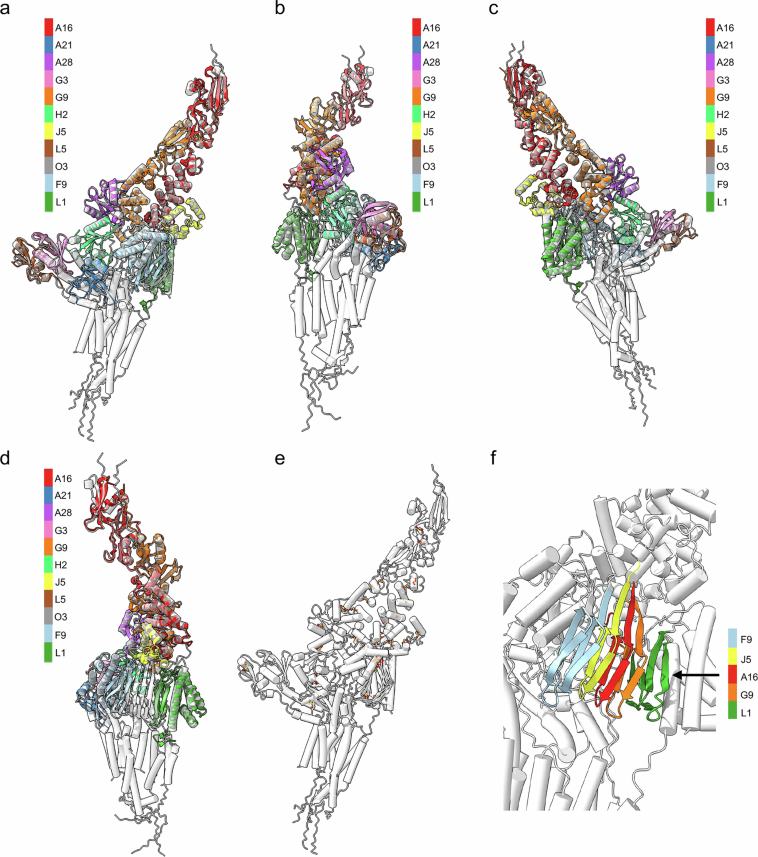
Correlation of predicted and experimental structures. **a-d**. The predicted holocomplexes in light gray with superimposed ectodomain structures from the Protein Data Bank (PDB) are shown in four 90° rotations. Individual ectodomains are identified by color. **e**. Close association of cysteine residues in holocomplex model. Pairs of cysteines in red. **f**. Aligned β-sheets of A16, G9, J5, F9 and L1 in AlphaFold3 EFC model.

We sought to determine whether binding and neutralization are related to the locations of proteins within the EFC. As neither the pre-fusion nor the post-fusion EFC structures had been determined experimentally at the time of this study, we used AlphaFold3 to model the 9-protein core and the 11-protein holocomplex (Fig. 3a–c). Computational methods including the predicted local distance difference test and predicted aligned error provided high confidence overall (Extended Data Fig. 5a–d), although the placement of L1 relative to the other subunits had a higher predicted error. The available ectodomain structures from the PDB could be superimposed onto the holocomplex model (Extended Data Fig. 6a–d); the root mean square deviation of the pruned structural alignments ranged from 0.473 to 1.07 Å (Supplementary Data 1) indicating good correspondence. Furthermore, 33 of the 34 known disulfide-bonded sulfur atom pairs were positioned within 2.15 Å of each other (Extended Data Fig. 6e), well within the distance needed to form disulfide bonds^19^. In addition, the predicted adjacency of external domains of A16 and G9, A28 and H2, and L5 and G3 in the model is consistent with experimental data demonstrating that these pairs form stable heterodimers^12,17,20^.

From the network diagram (Fig. 3d), based on the buried solvent-accessible areas between pairs of proteins, three groups were predicted within the core structure of the EFC: G3, L5, A21 and O3 form the base and interact with H2 and A28, which serve as a bridge to the apical proteins G9, A16 and J5. We determined that A28 but not H2 interacts directly with A16/G9 by immunoprecipitation with soluble proteins (Extended Data Fig. 2f). G9, A16 and J5 are also predicted to interact extensively. L1 and F9 appear to make multiple contacts with other EFC proteins. Additional structural features of the model include the close packing of all transmembrane domains and the alignment of parallel β-sheets of A16, G9, J5, F9 and L1 to form a hinge-like structure (Extended Data Fig. 6f) that could modulate structural transitions. Supplementary Video 1 shows the surface lipophilicity and the parallel β-sheets of a three-dimensional space-filling model of the holocomplex.

Next, we mapped the regions of the EFC on the AlphaFold3 model that are targets of neutralizing and non-neutralizing antibodies. The proteins that are targets of neutralizing antibodies are shown in colours, whereas the non-neutralizing region comprising the base proteins L5, A21, G3, O3 and the associated bridging H2 protein is depicted in grey rotated in two dimensions (Fig. 3e,f) and in three dimensions (Supplementary Video 2). The neutralizing antibody targets comprise A16, G9 and J5 at the apex of the EFC, and A28, F9 and L1 positioned below. The grouping of the non-neutralizing proteins G3, L5, A21 and H2 near the base suggests that this region may be obscured by other proteins in the viral membrane.

### Neutralizing antibodies to G9 and A16 correlate with protection

**Fig. 4:**
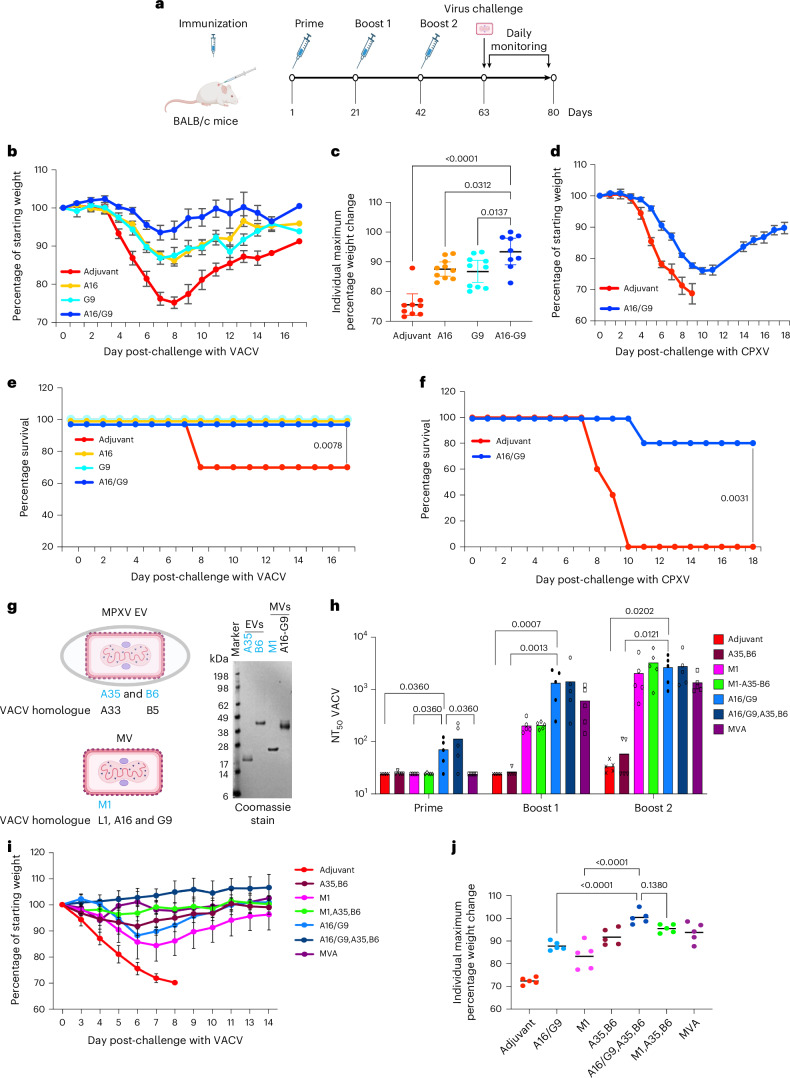
Protection of A16-, G9- and A16/G9-immunized mice against VACV and CPXV infections. **a**, Female BALB/c mice from two independent experiments (*n* = 10 per group) were primed and boosted twice with 10 µg of A16, G9 or A16/G9 proteins plus AddaVax or with 10^7^ p.f.u. of MVA before challenge intranasally with 10^5^ p.f.u. of VACV-WR. **b**, The percentage of starting weight post-VACV challenge. Geometric mean values ± s.d. are shown. **c**, The percentage maximum weight change of individual mice. Bars represent geometric means. Significance by one-way ANOVA with Dunnett’s multiple comparisons. **d**, The percentage of starting weight following challenge with 10^6^ p.f.u. of CPXV (*n* = 5). Geometric mean values ± s.d. are shown. **e**, Survival of VACV challenge. Significance was determined by a Kaplan–Meier log-rank test. **f**, Survival following CPXV challenge. Significance determined as in **e**. **g**, A diagram of EV and MV showing the associated proteins and Coomassie blue-stained SDS–PAGE gel of secreted A35, B6, M1 and A16/G9 purified with Ni–NTA beads. Representative of repeated experiments. **h**, Groups of female mice (*n* = 5) were primed and boosted twice with 10 µg of recombinant MV and EV proteins plus AddaVax, or 10^7^ p.f.u. of MVA and anti-VACV-neutralizing titre of serum from individual mice were determined after priming and boosts. Significance by one-way ANOVA with Dunnett’s multiple comparisons. **i**, The percentage of starting weights following challenge with 10^6^ p.f.u. of VACV. Immunogens are indicated on the right. Geometric mean values ± s.d. are shown. **j**, The percentage maximum weight change of individual mice. Bars indicate geometric means. Significance between groups was determined as in panel **c**. Experiments in panels **g** and **h** were repeated with similar results. Schematic in **a** and **g** created in BioRender; Yu, H. https://biorender.com/fmctsd0 (2026).

Mice primed and boosted with A16, G9 or A16/G9 (Fig. 4a) were challenged intranasally with 10^5^ plaque-forming units (p.f.u.) of VACV WRvFire, which expresses firefly luciferase allowing bioluminescent imaging (BLI) of virus replication and spread^21,22^. Immunized mice maintained a higher weight than the control mice, with those receiving A16/G9 having the least weight loss (Fig. 4b). A comparison of the maximum per cent weight change of individual mice confirmed that optimal protection was achieved following immunization with A16/G9 (Fig. 4c). All A16-, G9- or A16/G9-immunized mice survived compared with 70% of control mice (Fig. 4e). Thus, the immunity conferred by A16/G9 as well as A16 or G9 was sufficient to reduce weight loss and prevent lethality.

**Extended Data Fig. 7:**
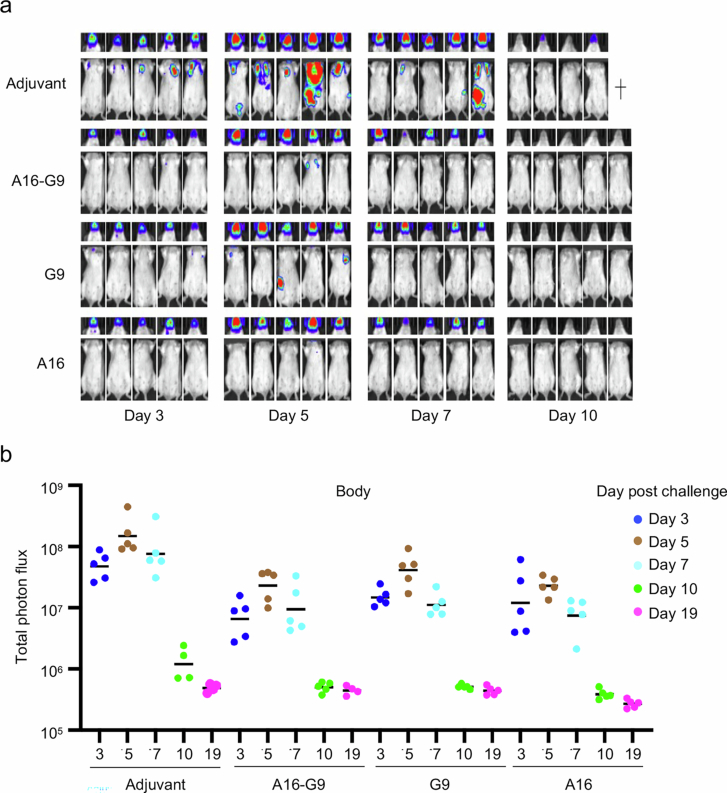
Bioluminescence imaging. Female BALB/c mice were anesthetized with isoflurane and injected intraperitoneally (IP) with Xenolight d-luciferin substrate (150 μg/g body weight) 8 to 10 min before imaging with an IVIS Lumina LT series III system (Perkin Elmer, Waltham, MA) as described^21^. Luminescent exposures (1-60 s) were collected using small or medium binning factors and varied f-stop settings. Living Image Software (Perkin Elmer) facilitated acquisition and analysis. Photon flux was measured by outlining the head and body separately as regions of interest, quantifying light emission in photons per second per square centimeter per steradian. **a**. BLI of heads and bodies of immunized mice post-challenge with WRvFire from one of two similar experiments (n = 5) described in Fig. 4a. **b**. Photon flux of bodies of mice imaged in panel a. Bars represent means.

To evaluate WRvFire replication and spread during infection, luciferin was injected intraperitoneally, and BLI was measured daily in live mice. Owing to variations in light quenching by different tissues, BLI of head and body was determined separately in split images (Extended Data Fig. 7a). Mice immunized with A16, G9 or A16/G9 had BLI in the head that diminished faster than in the controls and few had discernible BLI in the chest or body, which was confirmed by quantitative photon flux measurements (Extended Data Fig. 7b).

To determine cross-OPXV protection, mice were primed and boosted with A16/G9 followed by a lethal 10^6^ p.f.u. CPXV challenge. Despite weight loss (Fig. 4d), 80% of immunized mice survived compared with complete mortality in the control group, indicating cross-protection (Fig. 4f).

Additional experiments were carried out to determine whether immunization with the MPXV EV proteins A35 and B6 together with A16/G9 would provide more complete protection against elevated VACV challenge doses, as had been observed when these EV proteins were combined with the MPXV M1 MV protein in mRNA^23^ and virus-like particle^24^ vaccines. SDS–PAGE analysis of the MV and EV proteins is shown in Fig. 4g. Mice were primed and boosted with A35 + B6, M1, M1 + A35 + B6, A16/G9, A16/G9 + A35 + B6, or adjuvant alone as a negative control or MVA as a positive control (Fig. 4h). After the prime and first boost, A16/G9 induced significantly higher neutralizing antibodies than M1, although the difference diminished after the second boost. As A35 and B6 are EV proteins, their addition did not affect the neutralizing activities of M1 and A16/G9. Nevertheless, antibodies to EV proteins contribute to protection by reducing virus spread. A lethal dose of VACV was used for challenge of mice that were primed and boosted twice to better discern additive effects of the immunization with multiple proteins. Each of the immunized groups lost less weight and survived compared with the controls, which all succumbed. However, only the A16/G9 + A35 + B6 group maintained and increased weight over the course of the experiment (Fig. 4i). A35 + B6 significantly enhanced protection when combined with either M1 or A16/G9 (Fig. 4j). However, the difference between A16/G9 + A35 + B6 and M1 + A35 + B6 was not significant.

### Antibodies binding to A16 and G9 are rarely detected following OPXV infections

**Fig. 5:**
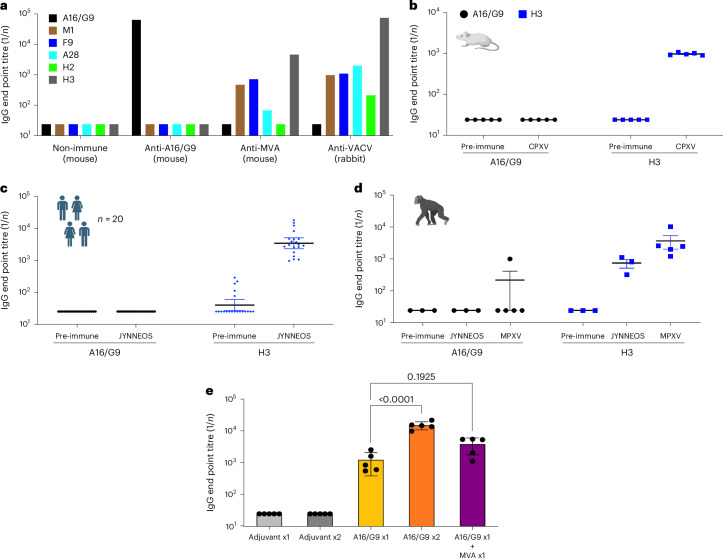
Limited antibodies to A16 and G9 in sera following VACV, CPXV and MPXV infections. **a**, Pooled sera from ten non-immunized, ten A16/G9 immunized, ten MVA immunized female mice and rabbit anti-VACV IgG (batch 304680, Quality Biologicals) were analysed by ELISA to VACV G9, A16, F9, A28, H2 and MPXV M1 and H3 (homologues of VACV L1 and H3, respectively) proteins. **b**, ELISA to A16/G9 and H3 antigens in serum from individual female mice (*n* = 5) primed and boosted percutaneously with CPXV. **c**, ELISA to A16/G9 and H3 antigens in sera from individual humans (*n* = 20) primed and boosted with the JYNNEOS vaccine subcutaneously. **d**, ELISA to A16/G9 and H3 antigens in sera from individual rhesus macaques primed and boosted with the JYNNEOS vaccine (*n* = 3) subcutaneously or infected with MPXV (*n* = 5) intravenously. **e**, Five groups of female mice (*n* = 5) were immunized according to the following protocol: group 1 received adjuvant only on day 0 (adjuvant x1); group 2 received adjuvant on days 0 and 21 (adjuvant x2); group 3 received 10 µg of A16/G9 plus AddaVax on day 0 (A16/G9 x1); group 4 received 10 µg of A16/G9 plus AddaVax on days 0 and 21 (A16/G9 x2); and group 5 received 10 µg of A16/G9 plus AddaVax on day 0 and 10^7^ p.f.u. of MVA on day 21 (A16/G9 xMVA). Mice were bled on day 31 and binding to A16/G9 was determined by ELISA. Mean values ± s.e.m. shown in **c**–**e**. Significance was determined by one-way ANOVA with Dunnett’s multiple comparisons. Experiments in **a**, **b**, **d** and **e** were repeated with similar results. Schematic in **b**–**d** created in BioRender; Yu, H. https://biorender.com/fmctsd0 (2026).

Antibodies to A16/G9 were not detected in sera from mice and rabbits infected with MVA or the pathogenic replicating strain of VACV, whereas antibodies to the H3 cell attachment protein as well as other EFC proteins (for example, M1, F9, A28 and H2) were detected (Fig. 5a). Similarly, mice immunized with CPXV made antibodies to H3 but not to A16/G9 (Fig. 5b). Furthermore, human subjects^25^ and rhesus macaques^26^ primed and boosted with the JYNNEOS MVA vaccine made detectable antibodies to H3 but not to A16/G9 (Fig. 5c,d). Only one of five MPXV-infected macaques made anti-A16/G9 binding antibodies (Fig. 5d). An additional experiment demonstrated that immunization with MVA did not boost production of anti-A16/G9 antibodies to a significant extent in mice that had been primed with soluble A16/G9 protein compared with a second immunization with A16/G9 (Fig. 5e). Thus, antibodies to A16/G9 were largely undetected following OPXV infections.

### Overcoming suppression of A16/G9 immunogenicity

**Fig. 6:**
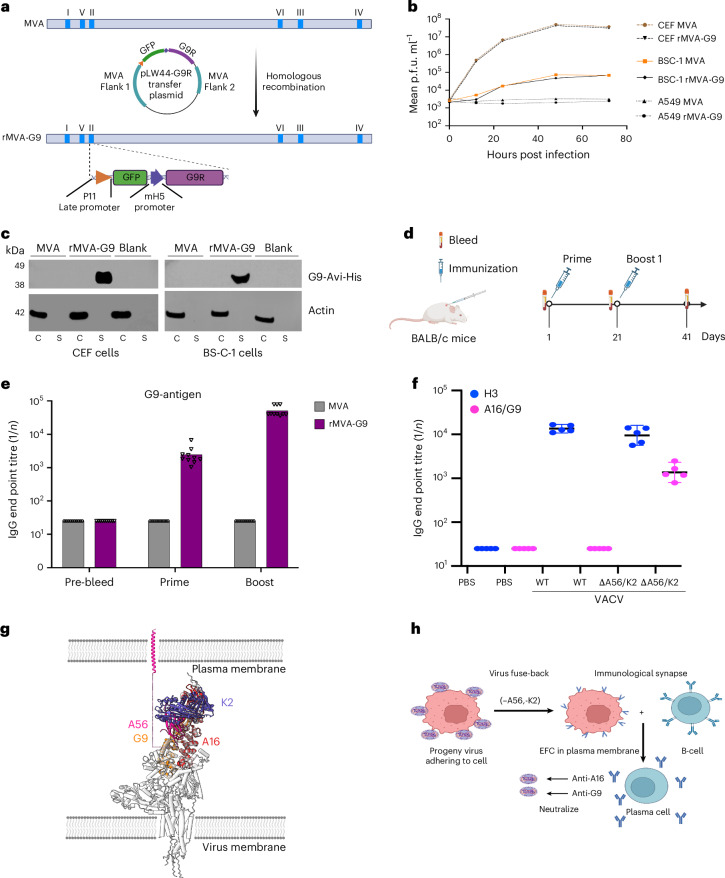
Enhanced antibody responses by recombinant VACVs that secrete G9 or have A56/K2 gene deletions. **a**, Construction of recombinant MVA (rMVA-G9) by homologous recombination. The six deletion sites in the MVA genome are indicated by Roman numerals. DNA encoding the secreted form of G9-Avi-His and GFP was inserted into the LW44 shuttle vector under control of the mH5 early/late promoter, which allowed homologous recombination into deletion II and selection of plaques with green fluorescence. **b**, Replication of parental MVA and rMVA-G9 in permissive CEF, semi-permissive BS-C-1 and non-permissive A549 cells. Mean values of three technical replicates are shown. **c**, Immunoblot analysis of G9-Avi-His in the cell lysates and medium of BS-C-1 and CEF cells infected with parental MVA or rMVA-G9. **d**, Immunization scheme of BALB/c mice (*n* = 10) infected with 10^7^ p.f.u. of parental MVA or rMVA-G9. **e**, Analysis of anti-G9 antibodies by ELISA in the serum of individual immunized mice (*n* = 10). **f**, Induction of antibodies to A16/G9 by A56/K2 gene deletion VACV (ΔA56/K2). Female BALB/c mice (*n* = 5) were immunized percutaneously with wild-type (WT) VACV-WR or ΔA56/K2. Sera were collected after 3 weeks and ELISAs performed with H3 and A16/G9 antigens. Mean values ± s.e.m. **g**, Experimentally determined A16/G9 and A56/K2 ectodomains^27^ overlaid on AlphaFold3 EFC manually linked to viral membrane at bottom and to plasma membrane with AlphaFold3 predicted A56 helical domain. **h**, A model for activation of A16/G9-specific B cells by ΔA56/K2 infection. In the absence of A56/K2, progeny virus particles fuse back to parental cells thereby inserting and exposing the EFC in the plasma membrane. B cells are then activated by A16/G9 displayed in cell membrane and antibodies produced. Experiments in **c**, **e** and **f** were repeated with similar results. Schematic in **a**, **d** and **h** created in BioRender; Yu, H. https://biorender.com/fmctsd0 (2026).

To test whether the failure of live OPXVs to induce antibodies to A16 and G9 could result from masking of the antigens within the virus membrane, DNA encoding a truncated G9 with a signal peptide was inserted into MVA by homologous recombination to form rMVA-G9 as depicted in Fig. 6a. The native G9, which is required for infectivity, was unaltered. Replication of the parental MVA and rMVA-G9 was similar in permissive chicken embryo fibroblasts (CEF), semi-permissive BS-C-1 cells and non-permissive A549 cells (Fig. 6b). As anticipated, G9 was secreted into the medium following infection of CEF or BS-C-1 cells with rMVA-G9 (Fig. 6c). Moreover, antibodies binding to G9 were detected after priming and increased after boosting mice with rMVA-G9, whereas none were detected in the serum of mice infected with parental MVA (Fig. 6d,e). These data demonstrate that secreted G9 but not G9 embedded in the viral membrane can elicit antibodies.

The VACV A56/K2 protein heterodimer binds specifically to A16/G9 (ref. ^12^), raising the possibility that this interaction influences immunogenicity. The A56/K2 complex is inserted into the cell membrane late in infection where it binds to A16/G9 of progeny virus particles on the cell surface^12^ as depicted by overlaying A56/K2 structural data^27^ on the AlphaFold3 model of A16/G9 in Fig. 6g. In the absence of A56/K2, progeny virus particles fuse back and insert the EFC into the plasma membrane of the parental cells, causing extensive syncytia. Thus, A56/K2 acts as a fusion suppressor to prevent superinfection by progeny virus particles and cell–cell fusion. To determine the possible role of A56/K2 in restricting the immunogenicity of A16/G9, mice were infected with either parental VACV strain WR or with a ΔA56/K2 gene deletion mutant. Although all mice developed antibodies against H3, anti-A16/G9 antibodies were made only by mice infected with the ΔA56/K2 mutant (Fig. 6f). As prevention of A56/K2 expression specifically results in the insertion of EFC proteins into the plasma membrane, we propose that membrane exposure allows B cells to recognize A16/G9, leading to activation and A16/G9 antibody production, as depicted in Fig. 6h.

## Discussion

The large number of poxvirus proteins required for entry and fusion offers numerous potential targets for the host immune system. Of the 11 EFC proteins, six induced neutralizing antibodies. These targets were located in the apical part of the EFC model generated by AlphaFold3. This model was constructed with the assumption of a single copy of each EFC component. In our AlphaFold3 analysis, G3, L5, A21 and O3 form the base, with H2 and A28 bridging to the apical A16, G9 and J5. This overall arrangement agrees with a cryo-electron microscopy structure that has two copies of the base and bridging proteins^10^.

We focused on the A16/G9 heterodimer, which is displayed at the apex of the EFC. Individually and as a heterodimer, the A16/G9 proteins induced cross-neutralizing polyclonal antibodies in mice to VACV, CPXV and MPXV consistent with their >97% sequence identities (Extended Data Table 1). Meola and colleagues^27^ produced anti-A16/G9 VHH antibodies that neutralized VACV and MPXV. Anti-A16/G9 antibodies were shown in the latter study to inhibit syncytia formation and in our study to inhibit VACV entry either before or after cell binding. Elucidating the precise mechanism of inhibition will depend on the future determination of the mechanism of fusion and the identification of the putative fusion peptide within the multiprotein EFC.

Mice immunized with A16, G9 or the heterodimer survived lethal VACV infection, consistent with findings of Meola et al.^27^ for mice immunized with the heterodimer. In addition, we demonstrated cross-protection of mice against a lethal CPXV infection as well as complete protection against VACV-mediated weight loss by combining EV proteins with A16/G9 for immunization. Mice immunized with A16/G9 produced a significantly higher neutralizing antibody titre than did M1, an MPXV EFC protein used in mRNA^23^ and virus-like particle^24^ vaccines. Thus, A16 and G9 are excellent candidates for inclusion in new multicomponent OPXV vaccines.

The finding that antibodies binding to G9 and A16 were elicited by soluble proteins but were undetectable or minimal in the blood of mice, monkeys and humans that had been vaccinated with the JYNNEOS MVA vaccine or infected with pathogenic VACV, CPXV or MPXV was intriguing. The latter findings align with a recent report of low A16/G9 antibody levels elicited by MVA^27^ and by an earlier study of antibodies elicited by recipients of the DryVax smallpox vaccine^28^. Several factors may contribute to a generally low immune response to the EFC proteins, including the assembly of MVs in the cytoplasm resulting in the absence of EFC proteins displayed in the plasma membrane^29^, the shielding of MV proteins by the EV membrane^30^, and the 10–20-fold lower abundance of EFC proteins relative to the major attachment proteins (P. D. Gershon, personal communication). Nevertheless, following virus infection, antibodies against other EFC proteins such as L1, F9 and A28 were detected, indicating that additional factors are involved in limiting the immunogenicity of A16/G9. Unlike these other EFC proteins, A16 and G9 are not released from virus particles by incubation with reducing agent and non-ionic detergent, suggesting strong interactions with non-membrane proteins^31,32^. In addition, A16 and G9 can be chemically cross-linked to immunodominant H3, A25, D8, A26 and A27 in the viral membrane^33^. The ability of a recombinant MVA expressing a secreted form of G9 to induce antibodies is consistent with the idea that A16/G9 is sequestered from the immune system in virus particles.

The finding that deletion of the viral fusion suppressor proteins A56/K2 enabled the mutated VACV to induce A16/G9 antibodies is particularly intriguing. A56/K2 is predominantly synthesized late in infection and is inserted as a heterodimer into the EV and plasma membrane where it can bind to released progeny at the cell surface. A56/K2 can bind to the A16/G9 heterodimer but not to either A16 or G9 individually^12^, consistent with subsequent mutagenesis and structural studies^27,34,35^. Deletion of A56/K2 results in the fusion of progeny virions back into the parental cells with deposition of the EFC into the cell membrane, which enables syncytium formation^12,36^. These data suggest that B cells recognize A16/G9 in the plasma membrane but not in virus particles. Thus, A56/K2 has multiple roles in suppressing the immunogenicity of some EFC proteins as well as in preventing superinfection and syncytium formation. This mechanism may apply to other OPXVs, as homologues of A56 and K2 are conserved (Extended Data Table 1).

The ability of anti-A16/G9 antibodies to neutralize virus despite the low immunogenicity of the sequestered protein seems paradoxical. The difference between antigenicity and immunogenicity may have several explanations. The EFC may only be exposed transiently when the EV membrane is disrupted at the surface of cells^37^ or by complement-mediated lysis after binding of EV antibodies as shown in vitro^38^, which may be sufficient for antibody neutralization but not for recognition by B cell receptors in lymph nodes. Although antibodies and B cell receptors are both immunoglobulins, B cell receptors are membrane bound while antibodies are soluble. Owing to this difference, antibodies can interact with antigen in three dimensions, whereas B cell receptors have more limited mobility in only two dimensions^39^. The concealment of the EFC by an additional membrane in extracellular virus and the binding of fusion suppressors to A16/G9 constitute immune evasion mechanisms unique to poxviruses. For these reasons, recombinant mRNA and virus-like particle vaccines that elicit protective antibodies to multiple proteins including A16/G9 and attenuated live OPXVs that express secreted EFC proteins or that have deletions of the A56/K2 genes may have advantages over current vaccines.

Limitations of the study: this study did not investigate passive antibody transfer or the contribution of cell-mediated immunity to the observed protection, which will be important to address in future studies.

## Methods

### Biosafety

Experiments in this study were approved by the NIH Institutional Biosafety Committee under Registration ID 24-BMC-135 and 25-BMC-095 and followed the NIH Guidelines for Research Involving Recombinant or Synthetic Nucleic Acid Molecules and the 6th edition of Biosafety in Microbiological and Biomedical Laboratories^40^. Studies were carried out with VACV and CPXV and potentially infectious material derived from animals in NIH-approved BSL-2 and ABSL-2 containment facilities and with MPXV in an NIH/CDC-certified BSL-3 select agent facility by trained smallpox-vaccinated personnel who maintain annual safety training from the Division of Occupational Health and Safety, wear protective gear, work in certified class II laminar flow hoods and follow Division of Occupational Health and Safety-approved Standard Operating Procedures. Sera from JYNNEOS-vaccinated and MPXV-infected macaques were from animals housed at BIOQUAL where they were provided with care and enrichment in accordance with the BIOQUAL Institutional Animal Care and Use Committee (D16-00052) as described in ref. ^26^. Upon receipt at NIH, the infectivity of serum from MPXV-infected macaques was inactivated by heating at 60 °C for 20 min.

### Ethics

We received anonymized human serum samples from healthy 18–50-year-old vaccinia virus-naive uncompensated volunteers who received two vaccinations with MVA-BN^25^. The sera were a subset of those obtained with written informed consent in the study protocol (NCT05512949) and provided without specific gender or age information by the Emmes Group (Frederick, MD, https://theemmesgroup.com).

### Animal studies

Animal experiments and procedures adhered to protocols LVD29E and LVD12E approved by the NIAID Animal Care and Use Committee. Mice were housed in groups of five under controlled ambient temperature and humidity. Euthanasia was performed when animals exhibited a 30% loss of initial weight. Sample sizes of five to ten mice were chosen based on previous experiments. Mice were randomly assigned to groups and technicians who monitored weights and morbidity were blinded regarding group allocations. Male white New Zealand rabbits, 2–4-months old, 1.5–2.0 kg were immunized with purified proteins in Complete Freund’s adjuvant followed by boosting three times in incomplete Freund’s adjuvant at Cocalico Biologicals. Housing and procedures for rabbits were in accordance with Animal Welfare Assurance no. D16-00398 (A3669-01).

### Cells and virus

BS-C-1 (ATCC CCL-26) and HeLa S3 (ATCC, CCL-2.2) cells used for virus propagation and Expi293F suspension cells (A14527, Thermo Fisher Scientific) for protein expression were certified to be negative for mycoplasma. Virus was isolated from HeLa S3 cells infected with VACV-WR (ATCC VR-1354), VACV WRvFire^41^, VACV-WR Gauss-A4^42^, CPXV-Brighton-GFP^43^ and MPXV Z-1979-GFP^23,44^. MVA^45^ and rMVA-G9 were grown in CEF. Virus was purified by sedimentation through two 36% (w/v) sucrose cushions, followed by banding on a 25–40% (w/v) sucrose gradient^44^.

### Cloning, expression and purification of recombinant proteins

Expression of secreted protein ectodomains by baculoviruses and purification from serum-free medium have been previously described^46^. Modifications and expression of M5 and G2 are described in Extended Data Fig. 1 and of A16 and G9 in Extended Data Fig. 2 using the modified albumin signal peptide^47^.

### Immunoprecipitation

ChromoTek V5-Trap Agarose (v5ta, ChromoTek) or ChromoTek Flag-Trap Agarose (ffak, ChromoTek) were employed according to the manufacturer’s protocol.

### Western blot

Blots were incubated at 4 °C overnight in PBS-T with anti-V5 (ab184331, Abcam), anti-Flag (F1804, Sigma-Aldrich), anti-His (66005-1-Ig, Proteintech), anti-GAPDH (60004-1-Ig, Proteintech) or anti-actin (60009-1-Ig, Proteintech). After washing, the membranes were incubated with the appropriate secondary IRDye 680RD goat anti-mouse or anti-rabbit IgG (926-68070, Li-Cor Biosciences) or with IRDye 800CW goat anti-rabbit, anti-mouse IgG (926-32211, Li-Cor Biosciences) or with goat anti-mouse or anti-rabbit horseradish peroxidase (HRP) and scanned with an Odyssey infrared scanner (Li-Cor Biosciences) or Radiance ECL (Azure Biosystems).

### Immunofluorescence

BS-C-1 cells were infected with vF13-HA^18^ at a multiplicity of 3 p.f.u. per cell for 16 h at 37 °C, then washed and incubated for 1 h at room temperature with anti-B5 rabbit polyclonal, anti-HA tag rabbit polyclonal (BioLegend), anti-HA mouse monoclonal antibody (CST clone 6E2, #2367) and anti-A16/G9 mouse polyclonal antibodies in PBS containing 10% FBS. After washing, cells were incubated for 1 h at room temperature with Alexa Fluor Plus 488- or 594-conjugated goat anti-rabbit or anti-mouse IgG secondary antibodies (Thermo Fisher). Cells were then washed with PBS and fixed in 4% paraformaldehyde in PBS (pH 7.4) for 15 min at room temperature. For permeabilization, cells were fixed as described above and incubated for 10 min with PBS containing 0.25% Triton X-100, followed by washes with PBS. Immunostaining with primary and secondary antibodies was carried out as described above and fluorescence images were acquired with a Leica Thunder microscope.

### Immunization and challenge of mice

Female 6–7-week-old BALB/c mice (Taconic Biosciences) were injected subcutaneously with 10 µg of protein in 50 µl of PBS emulsified with 50 µl of AddaVax (InvivoGen) or intramuscularly with 10^7^ p.f.u. of MVA in PBS supplemented with 0.05% bovine serum albumin (BSA) at 3-week intervals. Tail-scratch immunization was performed using 10^6^ p.f.u. of virus in 10 μl. Mice were challenged intranasally with 10^5^ p.f.u. or 10^6^ p.f.u. of VACV strain WR, WRvFire or CPXV-Brighton after the third immunization. Daily weight measurements were recorded and mice were euthanized if they lost 30% of their initial body weight by ‘blinded’ animal care technicians.

### ELISA

For antigen-binding ELISA, VACV F9 (BEI Resources, NR-2626), MPXV H3 (Sino Biological, 40893-V08H1) and MPXV M1 (Sino Biological, 40904-V07H) were applied to 96-well plates (0.1 µg per well) in carbonate–bicarbonate buffer (pH 9.6). Biotinylated proteins were applied to streptavidin-coated 96-well plates (0.25 µg per well). After washing and blocking with Tween-20 and non-fat dry milk, the plates were incubated with eightfold dilutions of heat-inactivated serum for 1 h at room temperature, followed by goat anti-mouse, rabbit, monkey or human IgG-horseradish peroxidase antibody (Thermo Fisher) and developed with SureBlue TMB substrate (SeraCare). Absorbance was measured at 370 nm and 492 nm using a Synergy H1 plate reader with Gen5 analysis software (Agilent Technologies). End point titres were calculated using Prism.

Intact virus ELISA was carried out as above except that sucrose gradient-purified VACV was added to 96-well plates (10^6^ p.f.u. per well) in 0.05 M carbonate–bicarbonate buffer and incubated overnight at 4 °C. Unbound virus was removed and bound virus was inactivated with 2% paraformaldehyde. For dissociated virus ELISA, purified VACV (10^9^ p.f.u. per ml) was incubated with 1% SDS for 1 h at room temperature, diluted 50-fold in 0.05 M carbonate–bicarbonate buffer supplemented with 0.5% BSA and 100 µl aliquots added to plates and incubated overnight at 4 °C and processed as above.

### Neutralization assay

A 96-well plate flow cytometric assay was used to quantitatively assess VACV-WR, CPXV-Brighton and MPXV Z-1979 expressing Aequorea coerulescens GFP, following established procedures^48^. NT_50_ values, representing the serum dilution at which 50% neutralization occurred, were determined using Prism software (GraphPad/Dotmatics).

### Cell binding and entry assays

To investigate cell binding, VACV Gauss-A4 virions^41^ (5 p.f.u. per cell) were treated with mouse antisera or rabbit anti-VACV polyclonal IgG at room temperature for 30 min and incubated with HeLa S3 cells in a 96-well plate at neutral pH for 1 h at 4 °C. The cells were washed to remove unbound virus and lysed using 1× Cell Lysis Buffer (Thermo Scientific, Pierce Gaussia Luciferase Flash Assay Kit) for 15 min at room temperature. Luciferase activity was measured in a Berthold Sirius luminometer.

For the pre-attachment entry inhibition assay, diluted serum was incubated with purified WRvFire at room temperature for 30 min and the mixtures were added to HeLa S3 cells at 4 °C for 1 h. The cells were washed twice with cold PBS to remove unbound virions. The cells were then incubated with prewarmed medium, washed with PBS at 4 °C and treated with 1× Cell Culture Lysis Reagent (Promega) for 20 min at room temperature with gentle agitation. Following centrifugation, the lysates were mixed with substrate and luciferase activity was measured according to the Promega protocol and quantified using a Berthold Sirius luminometer (Berthold Detection Systems).

For the post-attachment inhibition entry assay, HeLa S3 cells in 96-well plates were incubated with purified WRvFire (3 p.f.u. per cell) at 4 °C for 30 min to allow attachment. The cells were washed twice with cold PBS to remove unbound virus and incubated with 50 μl of serially diluted immune or control mouse serum for 30 min at 4 °C. After an additional two washes with cold PBS, the cells were incubated at 37 °C for 2 h and luciferase activity determined as above.

### Recombinant MVA

The recombinant MVA expressing G9 (rMVA-G9) was constructed by homologous recombination. The modified G9R open reading frame was inserted into the pLW44 vector under the control of the synthetic promoter mH5 and the GFP reporter gene was regulated by the p11 promoter in the pLW44 vector for selection^49^. Virus was clonally purified by repeated isolation of green fluorescent plaques.

### Statistical analysis

Data were analysed using GraphPad Prism 10 software with statistical methods indicated in the figure legends.

### Reporting summary

Further information on research design is available in the Nature Portfolio Reporting Summary linked to this article.

## Supplementary information

Reporting Summary

Peer Review File

Supplementary Video 1 Three-dimensional model of EFC.

Supplementary Video 2 Targets of neutralizing antibodies.

Supplementary Data 1 RMSD of EFC model.

## Source data

Source Data Fig. 1 Numerical data for plots in workbook Excel file.

Source Data Fig. 2 Numerical data for plots in workbook Excel file.

Source Data Fig. 3 Unprocessed western blots.

## Data Availability

All data supporting the findings of this study are available within the Article and its Source data. Requested materials developed at NIH will be provided by B.M. (bmoss@nih.gov) upon signing an NIAID material transfer agreement and assurance that work will be done under appropriate biosafety conditions. Requests are typically fulfilled within 2 weeks. Source data are provided with this paper.

## Extended data

**Extended Data Table 1:** Percent identities of orthopoxvirus proteins

EFC proteins	VACV WR^a^	MVA^b^	CPXV BR^c^	MPXV Zaire^d^	VARV BSH75_banu^e^
A16 homologues	100	99.74	98.94	97.35	97.88
G9 homologues	100	99.41	97.65	98.53	98.82
L1 homologues	100	99.60	98.40	98.00	99.20
F9 homologues	100	100	98.59	99.06	97.64
J5 homologues	100	100	97.74	98.50	97.74
A28 homologues	100	99.32	97.95	95.89	97.26
H2 homologues	100	100	98.41	99.47	99.47
L5 homologues	100	99.22	99.22	99.22	98.44
G3 homologues	100	100	97.30	98.20	97.30
A21 homologues	100	100	98.30	98.30	98.30
Fusion suppressor proteins					
A56 homologues	100	98.73	82.97	93.65	84.54
K2 homologues	100	99.46	94.91	92.00	93.57

^a^VACV WR GenBank: AY243312.1

^b^MVA GenBank: AY603355.1

^c^CPXV BR GenBank: AF482758.2

^d^MPXV Zaire GenBank: HM172544.1

^e^VARV BSH75_banu GenBank: DQ437581.1.
