# Supplementary material for: Immunogenicity of poxvirus A16/G9 entry–fusion subcomplex and its restriction by A56/K2 protein informs vaccine design
Source: Nat Microbiol. 2026 Jun 10;11(7):2008–20. doi: 10.1038/s41564-026-02392-6 (PMC13323091; doi:10.1038/s41564-026-02392-6)

Main Fig. 1b

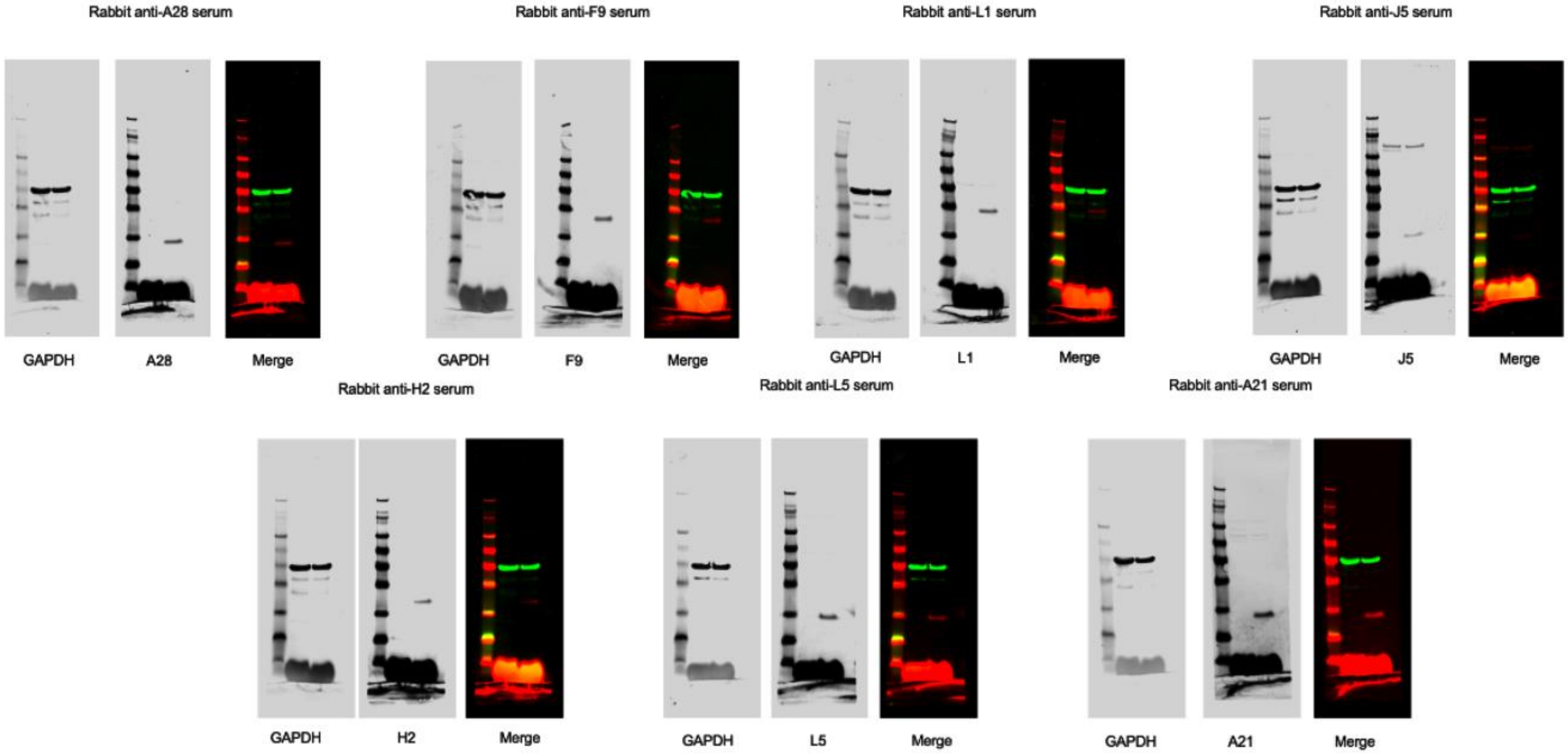
Rabbit anti-A28 serum
GAPDH
A28
Merge
Rabbit anti-F9 serum
GAPDH
F9
Merge
Rabbit anti-L1 serum
GAPDH
L1
Merge
Rabbit anti-J5 serum
GAPDH
J5
Merge
Rabbit anti-H2 serum
GAPDH
H2
Merge
Rabbit anti-L5 serum
GAPDH
L5
Merge
Rabbit anti-A21 serum
GAPDH
A21
Merge

Main Fig. 1h

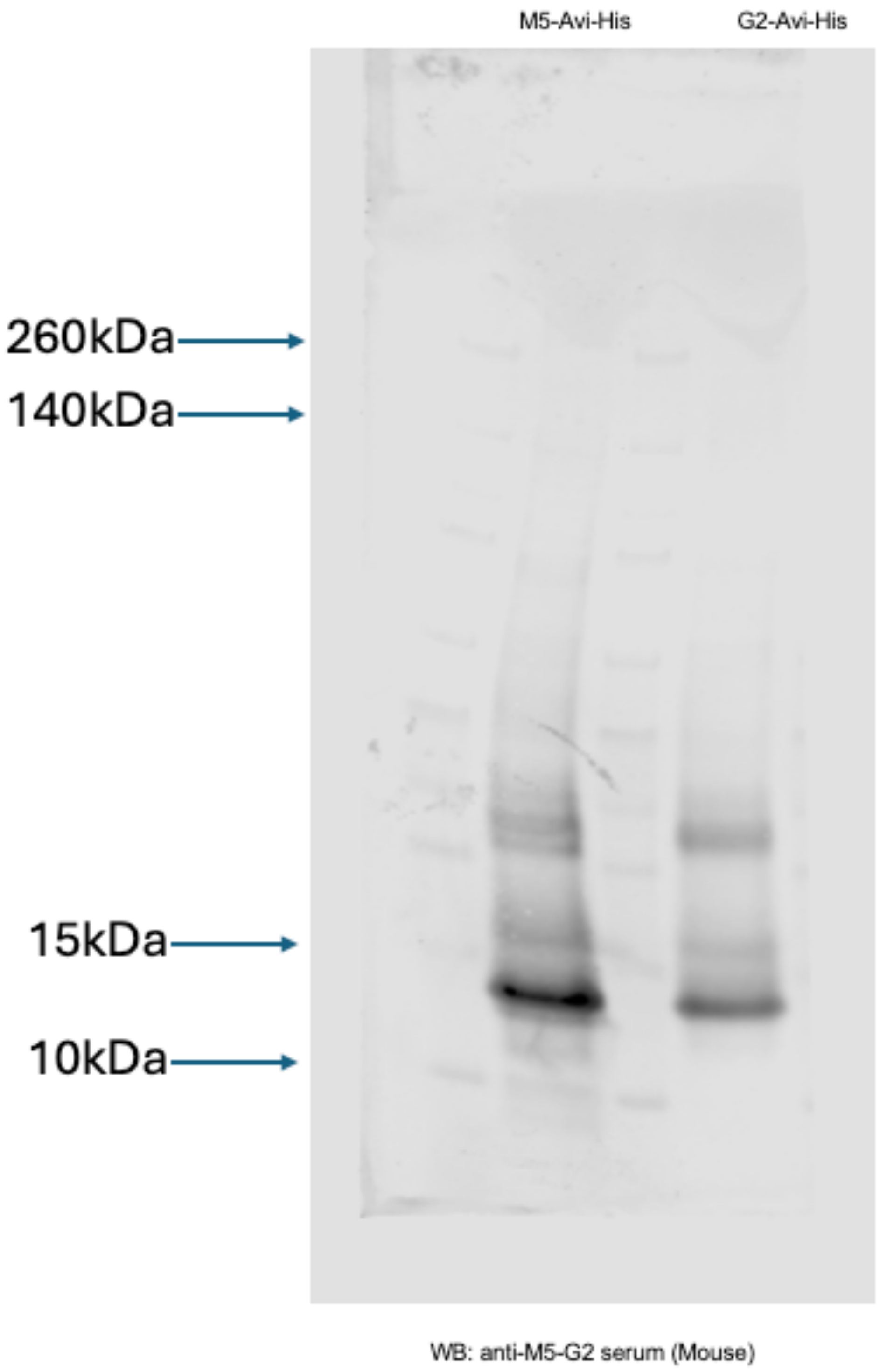
M5-Avi-His
G2-Avi-His
260kDa
140kDa
15kDa
10kDa
WB: anti-M5-G2 serum (Mouse)

Main Fig. 6c

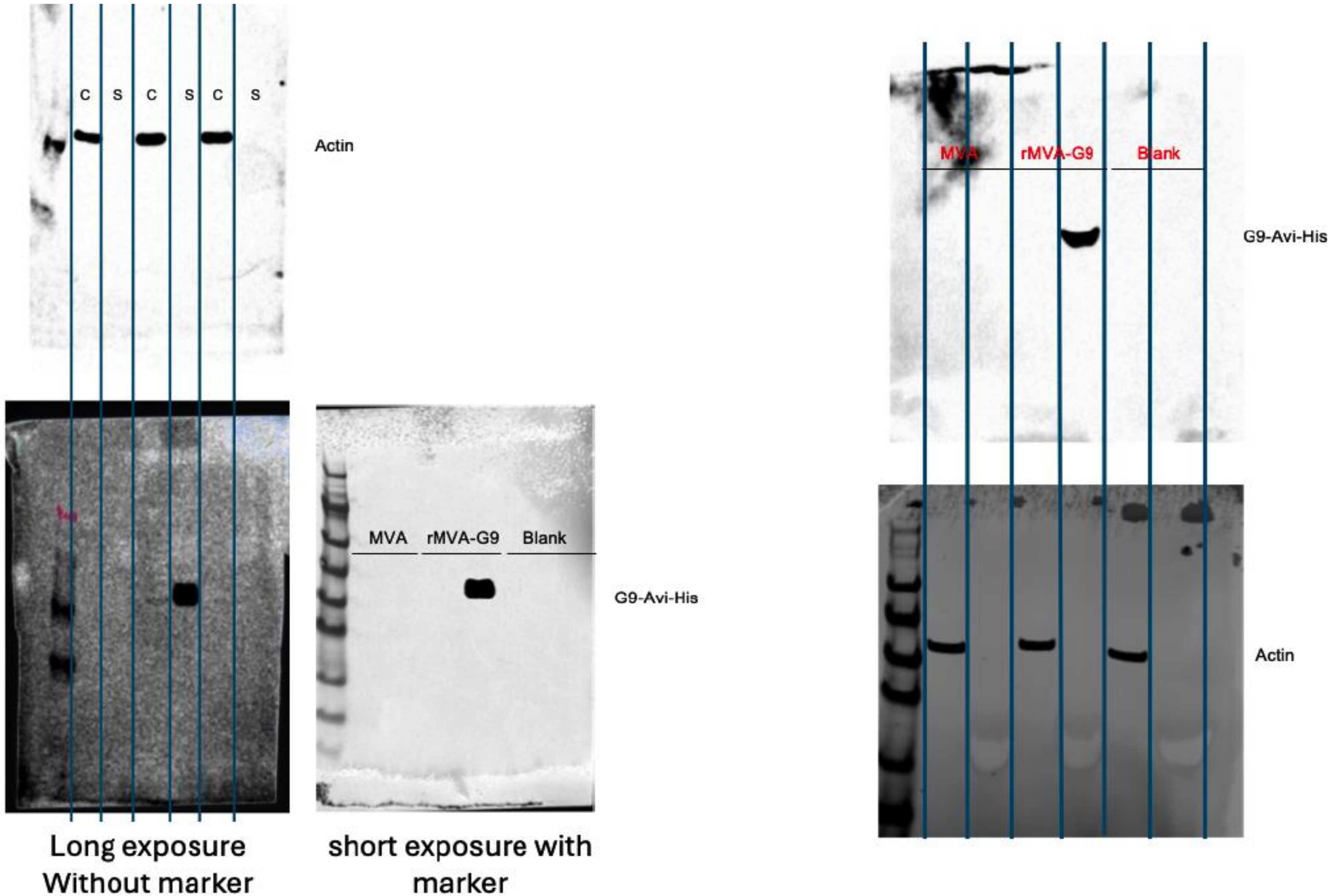

Long exposure Without marker

short exposure with marker

Extended Data Fig. 1a

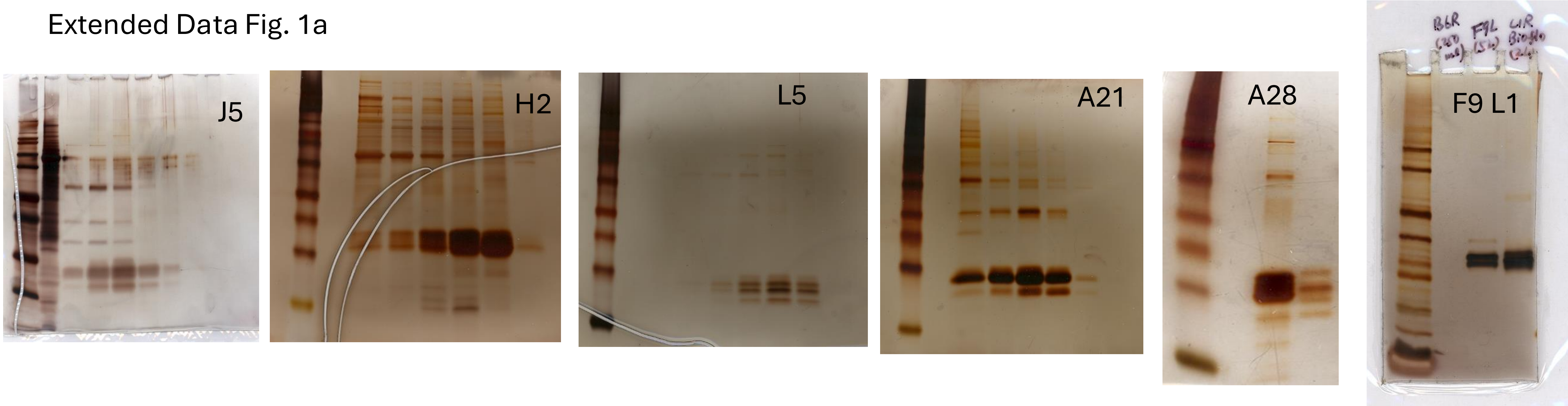

Extended Data Fig. 1d

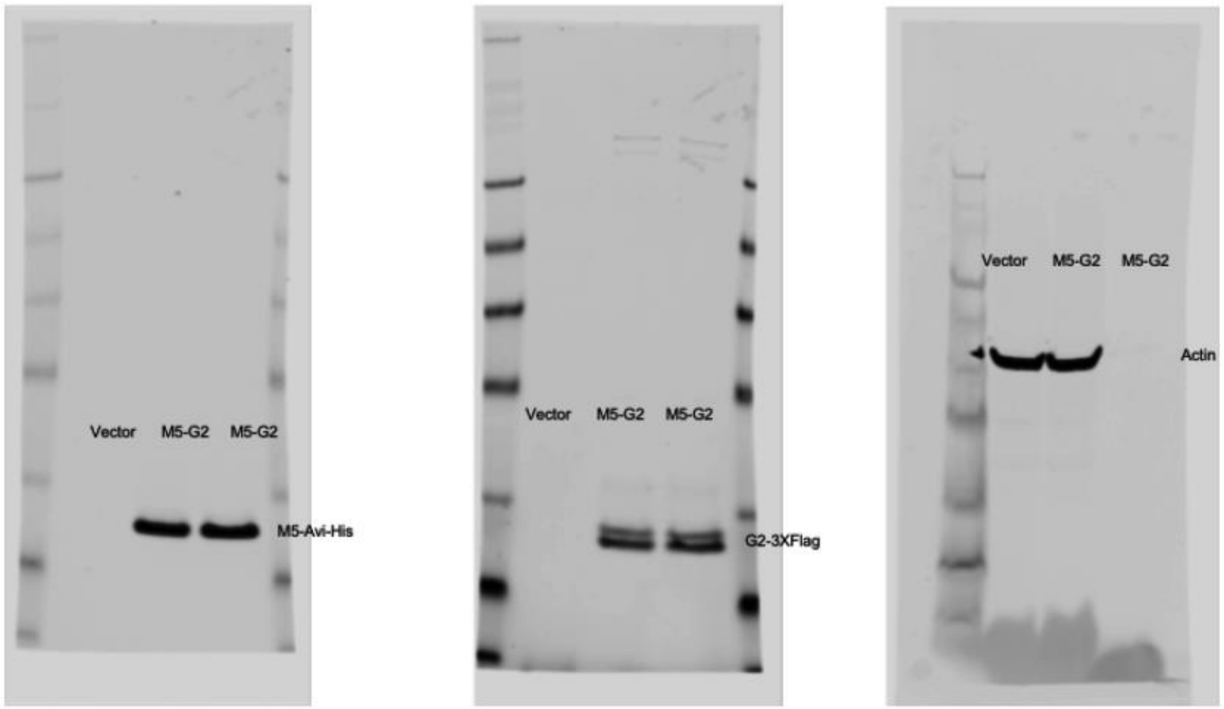

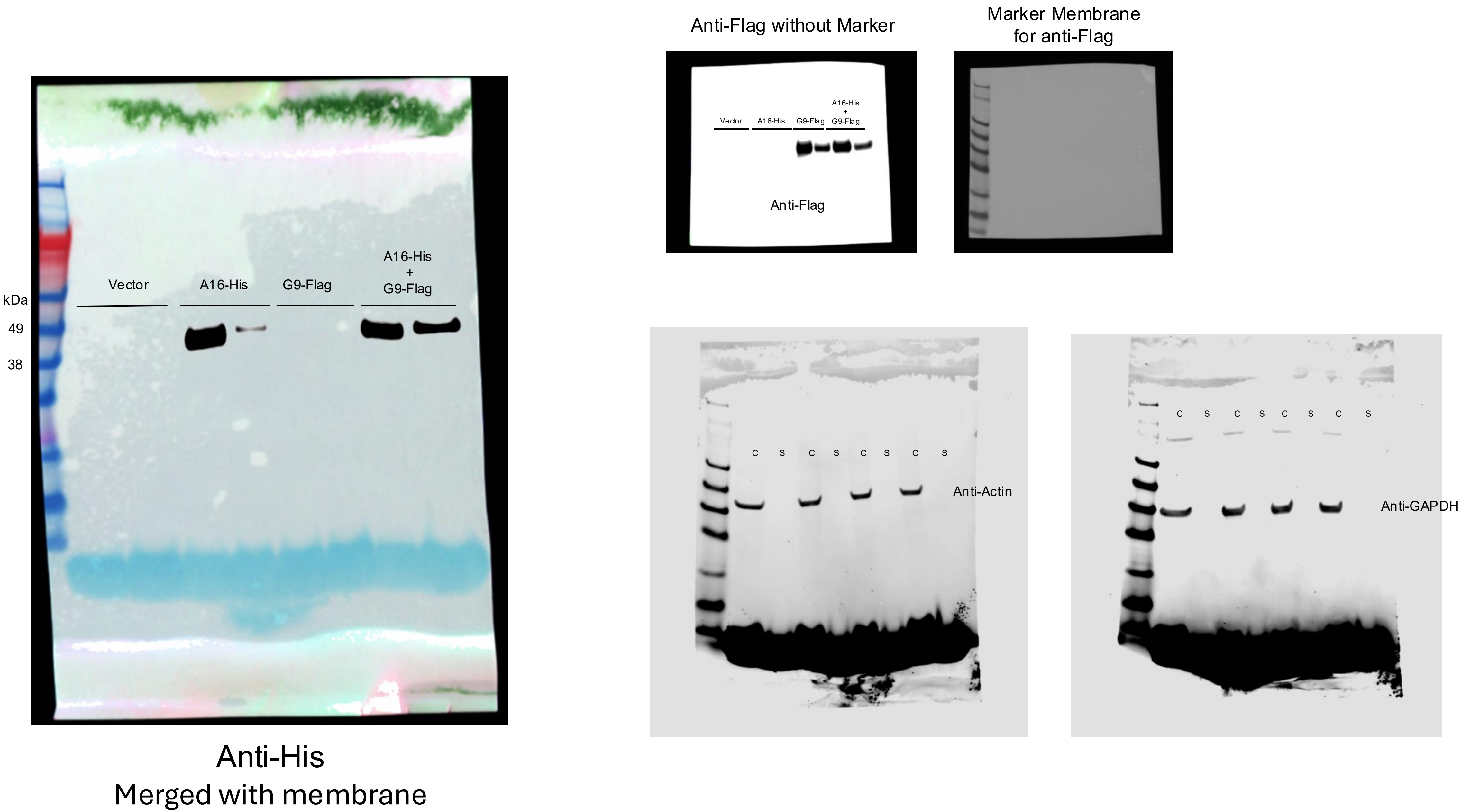

Extended Data Fig.2C

Extended Data Fig. 2d

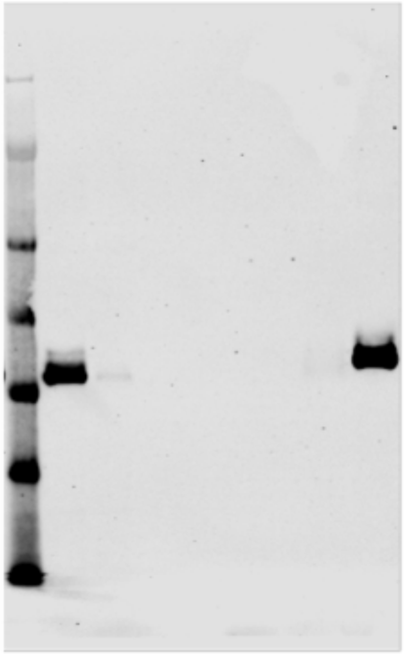

Anti-His Antibody

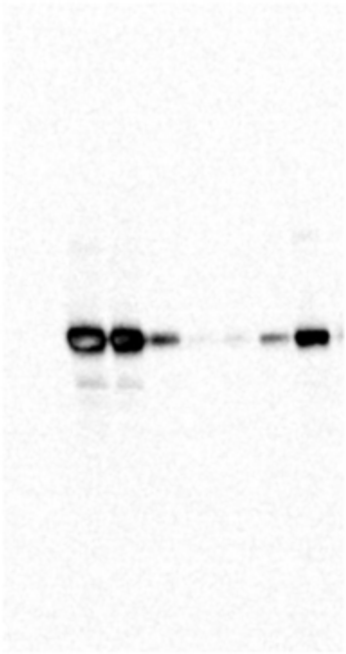

Anti-Flag Antibody

Extended Data Fig. 2e

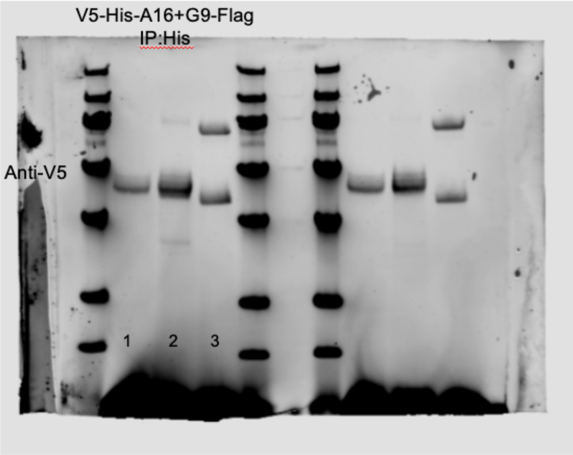

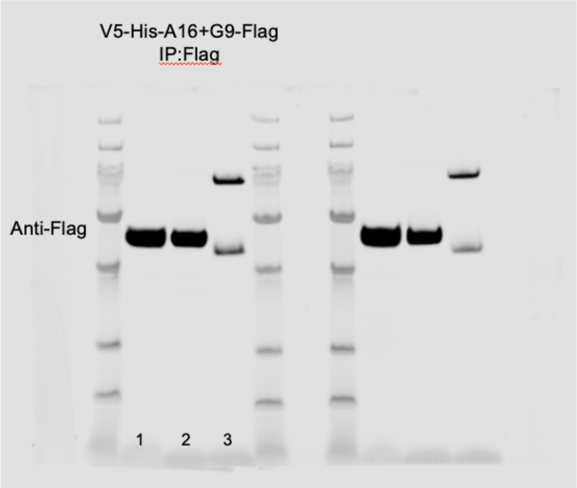

Key
1 Input + reducing
2 IP + reducing
3 IP - reducing

Extended Data Fig.2f

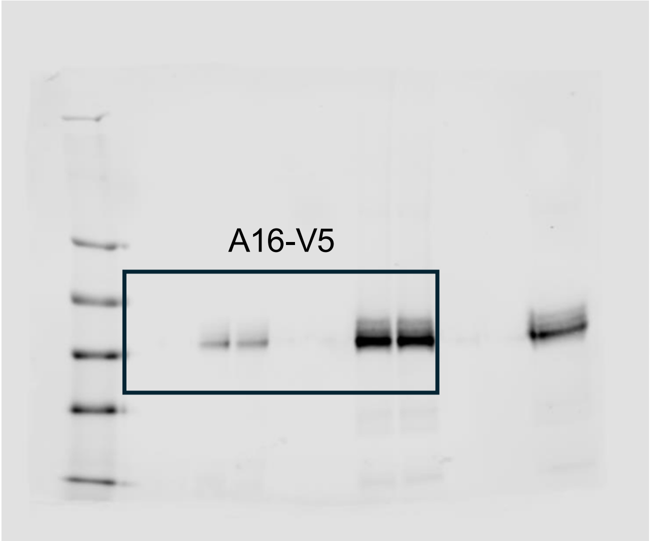

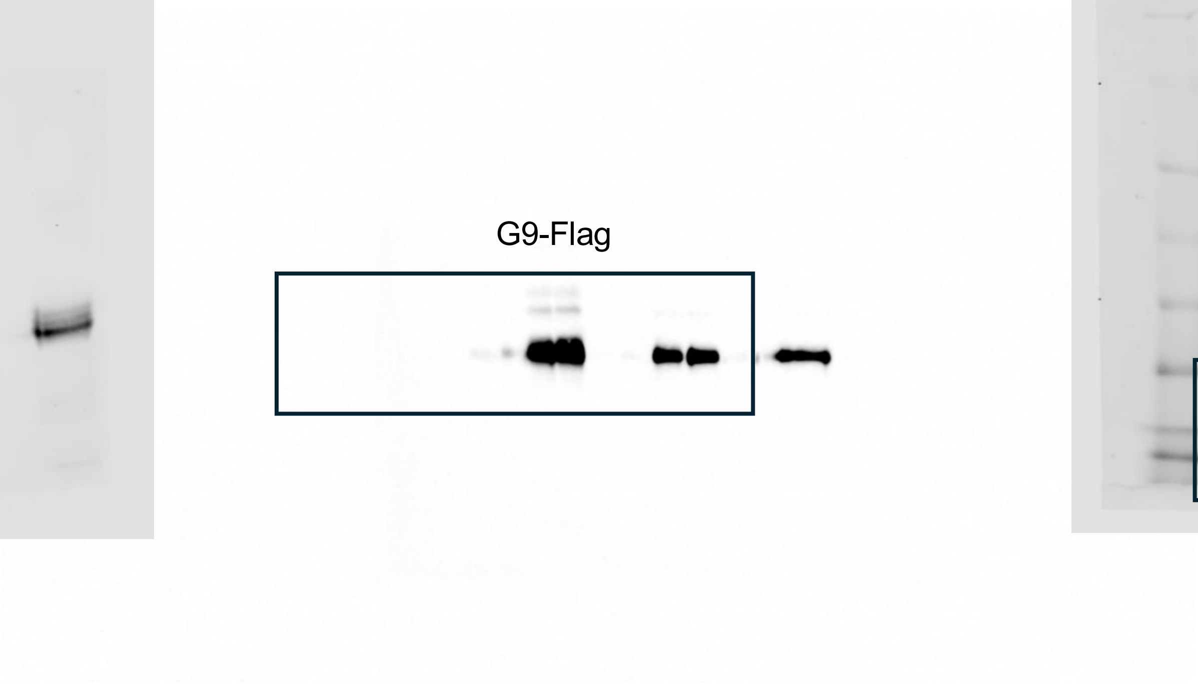

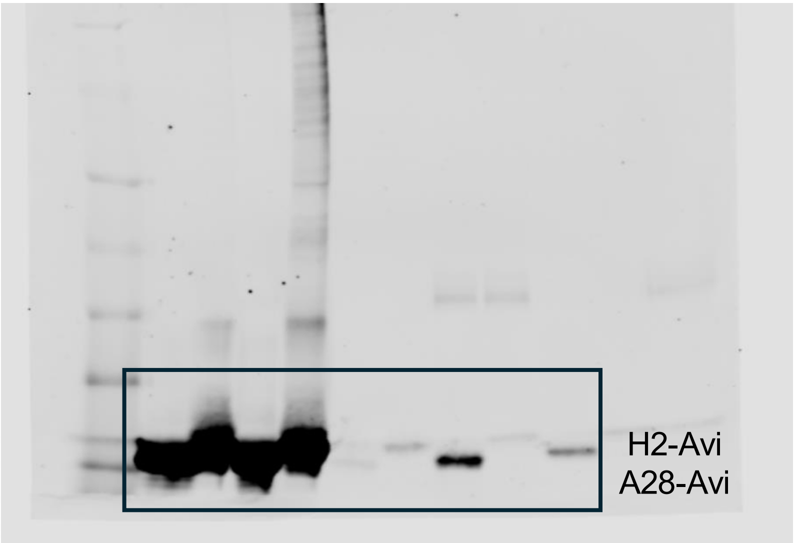

Supplement: Supplementary file 8 — Unprocessed western blots. [file 41564_2026_2392_MOESM8_ESM.pdf]
