# Supplementary material for: Immunogenicity of poxvirus A16/G9 entry–fusion subcomplex and its restriction by A56/K2 protein informs vaccine design
Source: Nat Microbiol. 2026 Jun 10;11(7):2008–20. doi: 10.1038/s41564-026-02392-6 (PMC13323091; doi:10.1038/s41564-026-02392-6)
Supplement: Supplementary file 2 — Peer Review File [file 41564_2026_2392_MOESM2_ESM.pdf]

nature portfolio

Peer Review File

Immunogenicity of poxvirus A16/G9 entry-fusion subcomplex and its restriction by A56/K2 protein informs vaccine design

Corresponding Author: Dr Bernard Moss

Version 0:

Reviewer comments:

Reviewer #1

(Remarks to the Author)
This manuscript by Yu and coworkers aimed to investigate the structure of the poxviral entry fusion complex (EFC) and its correlation with the activation of neutralizing and non-neutralizing antibodies against EFC proteins. Several EFC proteins have been identified as inducing neutralizing antibodies, while others do not activate neutralizing antibodies but rather bind to them. In doing so, they identified the G9/A16 EFC protein complexes as a potent activator of neutralizing antibodies that also cross-neutralize CPXV and MPXV. A16 and G9 proteins, positioned at the apex of a predicted AlphaFold3 EFC model, not only induce cross-neutralizing responses but also confer protection in murine models. Notably, these antigens appear to be immunologically silent during natural infection, suggesting a potential mechanism of immune evasion. Moreover, the effect of the G9/A16 heterodimer complex overcame the impact of the individual proteins G9 and A16, and also synergized with the effects of EV proteins A35 and B6. Together, these insights provide a refined structural and immunogenic map of the EFC, advancing the rational design of next-generation poxvirus vaccines and identifying another mechanism of poxviral-induced immune evasion, which may also contribute to the development of improved strategies against poxvirus.
In summary, the significance of the data presented in this manuscript is great. These data will be interest for poxvirus biology in general but also for translation. These fascinating data significantly enhance our understanding of poxvirus biology and the mechanism of the entry-fusion process from a fundamental research perspective. The manuscript substantially increases our understanding of the mechanism and structure of EFC. In addition, another mechanism of poxviral immune evasion has been identified, which may also contribute to understanding the pathogenesis of poxviruses. These data will then allow for the targeted development of antivirals and other preventive measures, particularly in the context of orthopoxvirus vaccine development, which is an urgent need at present due to the ongoing outbreaks of mpox in Africa. In doing so, this manuscript serves as an excellent example of how basic research knowledge can be translated into practical applications.
However, several points need more clarification and addition:
1. Mechanism of how antibodies to A16/G9 mediate neutralization and protection
- What is the explanation for the improved outcome of weight loss for the A16/G9 protein complex compared to the single proteins? Why is there no complete resolution of weight loss?
- Why can the A16/G9, G9, and A16 not inhibit the initial round of viral replication, as seen by the photon emission in the head and chest? Why is there no benefit seen in the A16/G9 as seen in the outcome of morbidity? Does this correlate with titers of VACV measured in the organs?
- How do the antibodies inhibit the viral spread?
- What, in contrast, is the protective mechanism of MVA? Are there any immunodominant proteins identified in MVA?
- Why is the combination with EV proteins not significantly better than the H3/EV combination when the single H3 versus the A16/G9 combination is considerably better? What is the mechanism of EV protein-mediated protection?
- Are the cross-neutralizing antibodies also protective?
- Can we correlate the activation of strong nAb to their function in the EFC? Can we relate this to the essential or non-essential roles of these proteins in EFC?
- If A16/G9 are blocked by nAb, can other EFC proteins compensate and further mediate fusion? Or is the conformation and functionality of EFC disturbed when single components are blocked?
- Is there a dosage effect of the A16/G9 protein immunization, such that if we increase the dosage, can we then block all EFCs initiated with a specific dosage of challenge virus?
- Can we induce the activation of A16/G9 antibodies after infection by using a kind of heterologous prime-boost vaccination strategy, that is, infection followed by protein-based vaccination with A16/G9? In a way similar to what is observed with the influenza virus and antigenic sin? Can a sublethal challenge dosage activate A16/G9 antibodies after infection?
- Is the very complex EFC itself also an immune evasion strategy when poxviruses apply more than 11 proteins for entry and fusion? At the same time, other viruses, such as measles or Coronaviruses, only use one protein to mediate fusion. Is the EFC also working when one or more of the proteins are inhibited? Is that the explanation for the partial replication that was detected after BLI? If it is blocked, is the complex structure an advantage or disadvantage?
2. A16/G9 immune evasion mechanism and impact on the EFC mechanism
- Is the immune evasion mechanism meaning that we did not detect A16/G9 ab after infection, also seen for MPXV infection? Are

human sera from MPXV-infected individuals also negative for A16/G9 antibodies? Is there a difference between lethal and non-lethal infection? In the different MPXV clades?
- What is the impact of the A56/K2 protein complex on the immune evasion mechanism of EFC? Is there an effect of syncytia formation, that is, is there activation of A16/G9-specific antibodies after syncytia formation? What is the outcome of infection with A56 KO Virus?
- How was the evolution of this immune evasion mechanism? Was this also present in VARV?
3. Correlates of Protection/Determinants of Vaccine-Induced Protection and Strategies for Broad Poxvirus Immunity
- If the A16/G9 are highly neutralizing, cross-reactive, and protective, but A16/G9 ab are not activated after MVA/VACV infection, what mediates the protection of MVA or VACV hyperimmune sera?
- What is the impact of the infection route for the activation of A16/G9 ab after infection with VACV or MVA?

- Can other EFC-proteins also mediate cross-protection?
- What is the impact of the entry mechanism used? Are there differences between endosome-mediated entry and fusion-mediated entry in the activation of nAb to EFC, and also in the activation of A16/G9 proteins?
- What is the impact of MVA dosages? Is the lower nAb also seen after 108 PFU immunization?
- What is the protective impact of a single vaccination versus a first boost versus a second boost? How is that correlated with MVA?
- What is the cross-neutralization of a single vaccination? In principle, this should be the same after the first vaccination?
- How could this be used to develop new antivirals for efficient blocking? Is this really effective, considering that inhibition of viral entry requires a very rapid application? how is that translated to the vaccine development? And how is the longevity?
- How can AlphaFold be used to predict other promising antigens for vaccine development further? How could that be applied to new emerging poxviruses?

Minor comments:
- Why are there different challenge dosages used?
- Ln 168 antibodies

Reviewer #2

(Remarks to the Author)
This manuscript presents a carefully executed study investigating the immunogenic and structural properties of the poxvirus entry-fusion complex (EFC), an 11-protein assembly required for viral entry. The authors use AlphaFold3 modeling and recombinant protein immunization to identify new neutralizing antibody targets, specifically A16 and G9. These targets are not immunogenic during live virus infection but induce protective immunity when delivered as recombinant proteins. The experimental breadth is impressive, and the work provides new biological insights and relevant translational applications for vaccine development. However, some areas require clarification, including the consistency of protocols, statistical reporting, the translational relevance of the findings beyond VACV, and the organization and clarity of the manuscript itself. Nonetheless, this is a strong and impactful manuscript that makes a valuable contribution to the field.
Strengths
• Important biological target: The EFC is central to viral entry and poorly characterized immunologically, making this work timely and valuable.
• Novelty: Identification of A16 and G9 as highly immunogenic and protective components, despite their invisibility in natural infection.
• Robust experimental design: Thoughtfully designed experiments, including immunization and challenge studies in mice, supported by bioluminescent imaging and neutralization assays.
• Strong integration of structural modeling: Use of AlphaFold3 to map immunogenic sites is innovative and well-executed.
• Clear vaccine relevance: Results provide rationale for improving orthopoxvirus vaccines beyond Jynneos, especially in the context of mpox.
Major concerns
1) Paradox of highly protective but poorly exposed antigens: One central conceptual gap that needs further discussion is the apparent paradox that A16 and G9 are highly protective vaccine targets, yet seem to be immunologically silent during natural infection. If these antibodies confer protection in vivo, this implies that the antigens must be exposed at some point, either on the virion surface or infected cells, to enable immune control.
The manuscript shows that recombinant A16 and G9 proteins induce potent neutralizing antibodies capable of inhibiting VACV, MPXV, and CPXV infection in vitro. In vivo, these antibodies confer protection against lethal VACV challenge, as demonstrated by reduced weight loss and improved survival in immunized mice. Structurally, AlphaFold3 modeling places A16 and G9 at the apex of the EFC, supporting the notion that they may be accessible on the virion surface. ELISA data suggest that antibodies against A16/G9 can bind to intact virions, although the extent of this binding is lower compared to other known immunogenic proteins.

However, despite these encouraging findings, the manuscript does not directly demonstrate surface exposure of A16 and G9 during natural infection. There is no imaging or biochemical evidence confirming their accessibility on native virions or infected cells. The relative abundance of A16/G9 compared to major immunodominant antigens (such as A27, H3, or L1) is also not assessed and indicated, leaving it unclear how frequently these proteins are encountered by the immune system. Moreover, the temporal dynamics of their expression or exposure during the viral life cycle are not explored, making it difficult to assess whether antibodies could engage their epitopes in a timely manner to mediate protection. While entry inhibition assays suggest functional blockade, the underlying mechanism—whether through binding to transiently exposed epitopes, structural interference within the EFC, or indirect effects—remains undefined. The lack of confirmatory surface staining of intact virions and absence of analysis of Fc-mediated effector functions (such as ADCC or phagocytosis) further limits the interpretation. These

gaps raise an important conceptual question: if these proteins are largely inaccessible during infection, how do vaccine-induced antibodies confer such robust protection? Additional experimental evidence is necessary to reconcile this paradox and fully establish the physiological relevance of A16 and G9 as vaccine targets.

2) Lack of consistent statistical reporting. While the text refers to p-values and statistical tests, the figures do not consistently include statistical annotations. In some key panels, there is no indication of which differences are significant. The authors should: Include statistical annotations (e.g., asterisks, exact p-values) on all figures where statistical tests were performed. Explicitly state in figure legends the statistical test used, sample size (n), and what comparisons were made.

3) Cross-protection claims should be nuanced. Although the paper frequently refers to orthopoxviruses broadly, all protection experiments were performed using VACV. Cross-neutralization of MPXV and CPXV are shown, but without in vivo validation. Since the authors show that neutralization does not always correlate with protection, the conclusion that these proteins confer cross-protection should be phrased more cautiously unless in vivo challenge data is provided for MPXV for example.

4) Immunization protocol transparency and consistency. The paper alternates between non-lethal and lethal VACV challenge models without clearly explaining the rationale. This lack of consistency makes interpretation harder. Clarifying why different challenge intensities were used and whether results are directly comparable would improve the narrative. Moreover, it is unclear whether: Protein doses were matched across all tested EFC components; Adjuvant concentration and formulation were standardized; Dosing schedules and number of boosts were consistent. The authors should clarify whether immunogenicity comparisons are valid across proteins.

Minor
Terminology: The manuscript repeatedly refers to “monkeypox” rather than “mpox,” which is now the WHO-recommended term. This should be corrected throughout to reflect current naming conventions.
Text redundancy and narrative flow: Flow of information can be difficult to follow; some critical experimental details or conceptual explanations are introduced only later in the manuscript.

Reviewer #3

(Remarks to the Author)
The authors present a manuscript entitled “Antibody Binding and Neutralizing Targets within the Predicted Structure of the Poxvirus Multiprotein Entry-Fusion Complex’ in which they identify neutralising antibody targets in the poxvirus entry complex and go on to use these targets to show that they might be good candidates for poxvirus vaccines.
Overall there are important observations in this paper, including the identification of A16 and G9 as targets of neutralising antibodies that can be induced by vaccination but not in the context of infection with whole virus. The authors also present a model of most of the entry complex using alpha fold 3 that sheds light on some of the entry complex function and how different types of antibody might interact with it. This manuscript has current relevance due to the requirement for better mpox vaccines. The manuscript is well written but there are however several points to be addressed before it is ready for publication. These are outlined below:

Figure 1: There should be measurements of protein purity, especially as proteins have been purified from serum-containing medium. Differences in purity will leads to differences in absolute mass of antigen injected into rabbits which can significantly influence the amount and quality of antibody generated, making comparisons between the sera generated against antigens hard to interpret.

Figure 1 C/D/E - are these data from single rabbits for each antigen? If so one recommendation is to use at least biological dupicates to be certain of the relative differences between antigens (especially considering the above comment about antigen purity).

Figure 2: The IPs (E and F) need a negative control protein eg. FLAG-GFP or a tagged non-binding VACV protein

Figure 3: It should be stated whether MVA expresses all the entry-fusion complex and whether there are major sequence differences in these proteins between VACV and MVA. Why are data from 3c using pooled sera and 3d using sera from individual mice? Figure 3F should include a positive control using a reagent that does inhibit VACV binding to cell surfaces.

Figure 5; The challenge experiments are convincing, but it would be helpful to link this protection to antibody function. Either the paper should acknowledge that protection can also be CD8 T-cell mediated and that the different vaccine antigens might elicit different CD8 memory responses (which is consistent with the data in 5C), or adoptive transfer experiments can be carried out to prove that the differences in protection levels between vaccine antigens, are at least in part, antibody-dependent (ie transferring serum from vaccinated mice into naive mice and challenging with VACV). Figure 5C the images are too small, suggest to move make them larger and move to supplementary data.

Figure 6: 6a, explain why pooled serum and how many were pooled in each case. The data presentation in 6a here is also confusing.

Figure 7: The model by Alphafold3 is interesting but its accuracy should be caveated in the text due to the lack of experimental validation. There are lots of protein/protein interaction predictions that could be tested with mutations to improve the confidence in what is otherwise a prediction with plenty of assumptions including the stoichiometry, the relative orientations, the degree flexibility of the individual chains etc… So either test the predictions or state that the assumptions that limit the confidence in accuracy.

Decision Letter:

3rd July 2025

*Please ensure you delete the link to your author homepage in this e-mail if you wish to forward it to your co-authors.

Dear Bernie,

Thank you for your patience while your manuscript "Antibody Binding and Neutralizing Targets within the Predicted Structure of the Poxvirus Multiprotein Entry-Fusion Complex" was under peer-review at Nature Microbiology. It has now been seen by 3 referees, whose expertise and comments you will find at the end of this email. Although they find your work of some potential interest, they have raised a number of concerns that will need to be addressed before we can consider publication of the work in Nature Microbiology.

In particular, all referees mention the need for deeper mechanistic insight into how antibody function leads to protection. Both referee #1 and #2 ask whether A16 and G9 are immunogenic in MPXV infected individuals. Specifically referee #2 requests to investigate why A16 and G9 are not immunogenic during virus infection but confer strong protection.

Should further experimental data allow you to address these criticisms, we would be happy to look at a revised manuscript. I should stress, however, that we would be reluctant to trouble our referees again unless we thought that their comments had been addressed in full.

We are committed to providing a fair and constructive peer-review process. Please do not hesitate to contact us if there are specific requests from the reviewers that you believe are technically impossible or unlikely to yield a meaningful outcome.

We strongly support public availability of data. Please place the data used in your paper into a public data repository, if one exists, or alternatively, present the data as Source Data or Supplementary Information. If data can only be shared on request, please explain why in your Data Availability Statement, and also in the correspondence with your editor. For some data types, deposition in a public repository is mandatory - more information on our data deposition policies and available repositories can be found at https://www.nature.com/nature-research/editorial-policies/reporting-standards#availability-of-data.

Please include a data availability statement as a separate section after Methods but before references, under the heading "Data Availability". This section should inform readers about the availability of the data used to support the conclusions of your study. This information includes accession codes to public repositories (data banks for protein, DNA or RNA sequences, microarray, proteomics data etc…), references to source data published alongside the paper, unique identifiers such as URLs to data repository entries, or data set DOIs, and any other statement about data availability. At a minimum, you should include the following statement: “The data that support the findings of this study are available from the corresponding author upon request”, mentioning any restrictions on availability. If DOIs are provided, we also strongly encourage including these in the Reference list (authors, title, publisher (repository name), identifier, year). For more guidance on how to write this section please see: http://www.nature.com/authors/policies/data/data-availability-statements-data-citations.pdf

If revising your manuscript:

* Include a “Response to referees” document detailing, point-by-point, how you addressed each referee comment. If no action was taken to address a point, you must provide a compelling argument. This response will be sent back to the referees along with the revised manuscript.

* If you have not done so already we suggest that you begin to revise your manuscript so that it conforms to our Article format instructions at http://www.nature.com/nmicrobiol/info/final-submission. Refer also to any guidelines provided in this letter.

* Include a revised version of any required reporting checklist. It will be available to referees (and, potentially, statisticians) to aid in their evaluation if the manuscript goes back for peer review. A revised checklist is essential for re-review of the paper.

When submitting the revised version of your manuscript, please pay close attention to our href="https://www.nature.com/nature-portfolio/editorial-policies/image-integrity">Digital Image Integrity Guidelines. and to the following points below:

-- that unprocessed scans are clearly labelled and match the gels and western blots presented in figures.
-- that control panels for gels and western blots are appropriately described as loading on sample processing controls
-- all images in the paper are checked for duplication of panels and for splicing of gel lanes.

EXTENDED DATA FIGURES

When re-submitting your manuscript, please ensure that any supplementary figures and tables that are crucial to the manuscript’s conclusions are converted into Extended Data figures and tables to increase visibility of these data. Extended Data figures and tables are online-only (present in the online PDF and full-text HTML versions of the paper), peer-reviewed display items that provide essential background to the article but are not included in the main article due to space constraints. A maximum of ten Extended Data display items (figures and tables) is permitted.

Finally, please ensure that you retain unprocessed data and metadata files after publication, ideally archiving data in perpetuity, as these may be requested during the peer review and production process or after publication if any issues arise.

Please use the link below to submit a revised paper:

Link Redacted

Note: This url links to your confidential homepage and associated information about manuscripts you may have submitted or be reviewing for us. If you wish to forward this e-mail to co-authors, please delete this link to your homepage first.

Nature Microbiology is committed to improving transparency in authorship. As part of our efforts in this direction, we are now requesting that all authors identified as 'corresponding author' on published papers create and link their Open Researcher and Contributor Identifier (ORCID) with their account on the Manuscript Tracking System (MTS), prior to acceptance. This applies to primary research papers only. ORCID helps the scientific community achieve unambiguous attribution of all scholarly contributions. You can create and link your ORCID from the home page of the MTS by clicking on 'Modify my Springer Nature account'. For more information please visit please visit www.springernature.com/orcid.

If you wish to submit a suitably revised manuscript we would hope to receive it within 6 months. If you cannot send it within this time, please let us know. We will be happy to consider your revision, even if a similar study has been accepted for publication at Nature Microbiology or published elsewhere (up to a maximum of 6 months).

In the meantime we hope that you find our referees' comments helpful.

Yours sincerely,

****

Reviewer Expertise:

Referee #1: Poxviruses, vaccines
Referee #2: Poxviruses, vaccines
Referee #3: Poxviruses, vaccines

Reviewer Comments:

Reviewer #1 (Remarks to the Author):

This manuscript by Yu and coworkers aimed to investigate the structure of the poxviral entry fusion complex (EFC) and its correlation with the activation of neutralizing and non-neutralizing antibodies against EFC proteins. Several EFC proteins have been identified as inducing neutralizing antibodies, while others do not activate neutralizing antibodies but rather bind to them. In doing so, they identified the G9/A16 EFC protein complexes as a potent activator of neutralizing antibodies that also cross-neutralize CPXV and MPXV. A16 and G9 proteins, positioned at the apex of a predicted AlphaFold3 EFC model, not only induce cross-neutralizing responses but also confer protection in murine models. Notably, these antigens appear to be immunologically silent during natural infection, suggesting a potential mechanism of immune evasion. Moreover, the effect of the G9/A16 heterodimer complex overcame the impact of the individual proteins G9 and A16, and also synergized with the effects of EV proteins A35 and B6. Together, these insights provide a refined structural and immunogenic map of the EFC, advancing the rational design of next-generation poxvirus vaccines and identifying another mechanism of poxviral-induced immune evasion, which may also contribute to the development of improved strategies against poxvirus.
In summary, the significance of the data presented in this manuscript is great. These data will be interest for poxvirus biology in general but also for translation. These fascinating data significantly enhance our understanding of poxvirus biology and the mechanism of the entry-fusion process from a fundamental research perspective. The manuscript substantially increases our understanding of the mechanism and structure of EFC. In addition, another mechanism of poxviral immune evasion has been identified, which may also contribute to understanding the pathogenesis of poxviruses. These data will then allow for the targeted development of antivirals and other preventive measures, particularly in the context of orthopoxvirus vaccine development, which is an urgent need at present due to the ongoing outbreaks of mpox in Africa. In doing so, this manuscript serves as an excellent example of how basic research knowledge can be translated into practical applications.
However, several points need more clarification and addition:
1. Mechanism of how antibodies to A16/G9 mediate neutralization and protection
- What is the explanation for the improved outcome of weight loss for the A16/G9 protein complex compared to the single proteins? Why is there no complete resolution of weight loss?
- Why can the A16/G9, G9, and A16 not inhibit the initial round of viral replication, as seen by the photon emission in the head and chest? Why is there no benefit seen in the A16/G9 as seen in the outcome of morbidity? Does this correlate with titers of VACV measured in the organs?
- How do the antibodies inhibit the viral spread?
- What, in contrast, is the protective mechanism of MVA? Are there any immunodominant proteins identified in MVA?
- Why is the combination with EV proteins not significantly better than the H3/EV combination when the single H3 versus the A16/G9 combination is considerably better? What is the mechanism of EV protein-mediated protection?

- Are the cross-neutralizing antibodies also protective?
- Can we correlate the activation of strong nAb to their function in the EFC? Can we relate this to the essential or non-essential roles of these proteins in EFC?
- If A16/G9 are blocked by nAb, can other EFC proteins compensate and further mediate fusion? Or is the conformation and functionality of EFC disturbed when single components are blocked?
- Is there a dosage effect of the A16/G9 protein immunization, such that if we increase the dosage, can we then block all EFCs initiated with a specific dosage of challenge virus?
- Can we induce the activation of A16/G9 antibodies after infection by using a kind of heterologous prime-boost vaccination strategy, that is, infection followed by protein-based vaccination with A16/G9? In a way similar to what is observed with the influenza virus and antigenic sin? Can a sublethal challenge dosage activate A16/G9 antibodies after infection?
- Is the very complex EFC itself also an immune evasion strategy when poxviruses apply more than 11 proteins for entry and fusion? At the same time, other viruses, such as measles or Coronaviruses, only use one protein to mediate fusion. Is the EFC also working when one or more of the proteins are inhibited? Is that the explanation for the partial replication that was detected after BLI? If it is blocked, is the complex structure an advantage or disadvantage?
2. A16/G9 immune evasion mechanism and impact on the EFC mechanism
- Is the immune evasion mechanism meaning that we did not detect A16/G9 ab after infection, also seen for MPXV infection? Are human sera from MPXV-infected individuals also negative for A16/G9 antibodies? Is there a difference between lethal and non-lethal infection? In the different MPXV clades?
- What is the impact of the A56/K2 protein complex on the immune evasion mechanism of EFC? Is there an effect of syncytia formation, that is, is there activation of A16/G9-specific antibodies after syncytia formation? What is the outcome of infection with A56 KO Virus?
- How was the evolution of this immune evasion mechanism? Was this also present in VARV?
3. Correlates of Protection/Determinants of Vaccine-Induced Protection and Strategies for Broad Poxvirus Immunity
- If the A16/G9 are highly neutralizing, cross-reactive, and protective, but A16/G9 ab are not activated after MVA/VACV infection, what mediates the protection of MVA or VACV hyperimmune sera?
- What is the impact of the infection route for the activation of A16/G9 ab after infection with VACV or MVA?

- Can other EFC-proteins also mediate cross-protection?
- What is the impact of the entry mechanism used? Are there differences between endosome-mediated entry and fusion-mediated entry in the activation of nAb to EFC, and also in the activation of A16/G9 proteins?
- What is the impact of MVA dosages? Is the lower nAb also seen after 108 PFU immunization?
- What is the protective impact of a single vaccination versus a first boost versus a second boost? How is that correlated with MVA?
- What is the cross-neutralization of a single vaccination? In principle, this should be the same after the first vaccination?
- How could this be used to develop new antivirals for efficient blocking? Is this really effective, considering that inhibition of viral entry requires a very rapid application? how is that translated to the vaccine development? And how is the longevity?
- How can AlphaFold be used to predict other promising antigens for vaccine development further? How could that be applied to new emerging poxviruses?

Minor comments:
- Why are there different challenge dosages used?
- Ln 168 antibodies

Reviewer #2 (Remarks to the Author):

This manuscript presents a carefully executed study investigating the immunogenic and structural properties of the poxvirus entry-fusion complex (EFC), an 11-protein assembly required for viral entry. The authors use AlphaFold3 modeling and recombinant protein immunization to identify new neutralizing antibody targets, specifically A16 and G9. These targets are not immunogenic during live virus infection but induce protective immunity when delivered as recombinant proteins. The experimental breadth is impressive, and the work provides new biological insights and relevant translational applications for vaccine development. However, some areas require clarification, including the consistency of protocols, statistical reporting, the translational relevance of the findings beyond VACV, and the organization and clarity of the manuscript itself. Nonetheless, this is a strong and impactful manuscript that makes a valuable contribution to the field.
Strengths
• Important biological target: The EFC is central to viral entry and poorly characterized immunologically, making this work timely and valuable.
• Novelty: Identification of A16 and G9 as highly immunogenic and protective components, despite their invisibility in natural infection.
• Robust experimental design: Thoughtfully designed experiments, including immunization and challenge studies in mice, supported by bioluminescent imaging and neutralization assays.
• Strong integration of structural modeling: Use of AlphaFold3 to map immunogenic sites is innovative and well-executed.
• Clear vaccine relevance: Results provide rationale for improving orthopoxvirus vaccines beyond Jynneos, especially in the context of mpox.
Major concerns
1) Paradox of highly protective but poorly exposed antigens: One central conceptual gap that needs further discussion is the apparent paradox that A16 and G9 are highly protective vaccine targets, yet seem to be immunologically silent during natural infection. If these antibodies confer protection in vivo, this implies that the antigens must be exposed at some point, either on the virion surface or infected cells, to enable immune control.

The manuscript shows that recombinant A16 and G9 proteins induce potent neutralizing antibodies capable of inhibiting VACV, MPXV, and CPXV infection in vitro. In vivo, these antibodies confer protection against lethal VACV challenge, as demonstrated by reduced weight loss and improved survival in immunized mice. Structurally, AlphaFold3 modeling places A16 and G9 at the apex of the EFC, supporting the notion that they may be accessible on the virion surface. ELISA data suggest that antibodies against A16/G9 can bind to intact virions, although the extent of this binding is lower compared to other known immunogenic proteins.

However, despite these encouraging findings, the manuscript does not directly demonstrate surface exposure of A16 and G9 during natural infection. There is no imaging or biochemical evidence confirming their accessibility on native virions or infected cells. The relative abundance of A16/G9 compared to major immunodominant antigens (such as A27, H3, or L1) is also not assessed and indicated, leaving it unclear how frequently these proteins are encountered by the immune system. Moreover, the temporal dynamics of their expression or exposure during the viral life cycle are not explored, making it difficult to assess whether antibodies could engage their epitopes in a timely manner to mediate protection. While entry inhibition assays suggest functional blockade, the underlying mechanism—whether through binding to transiently exposed epitopes, structural interference within the EFC, or indirect effects—remains undefined. The lack of confirmatory surface staining of intact virions and absence of analysis of Fc-mediated effector functions (such as ADCC or phagocytosis) further limits the interpretation. These gaps raise an important conceptual question: if these proteins are largely inaccessible during infection, how do vaccine-induced antibodies confer such robust protection? Additional experimental evidence is necessary to reconcile this paradox and fully establish the physiological relevance of A16 and G9 as vaccine targets.

2) Lack of consistent statistical reporting. While the text refers to p-values and statistical tests, the figures do not consistently include statistical annotations. In some key panels, there is no indication of which differences are significant. The authors should: Include statistical annotations (e.g., asterisks, exact p-values) on all figures where statistical tests were performed. Explicitly state in figure legends the statistical test used, sample size (n), and what comparisons were made.

3) Cross-protection claims should be nuanced. Although the paper frequently refers to orthopoxviruses broadly, all protection experiments were performed using VACV. Cross-neutralization of MPXV and CPXV are shown, but without in vivo validation. Since the authors show that neutralization does not always correlate with protection, the conclusion that these proteins confer cross-protection should be phrased more cautiously unless in vivo challenge data is provided for MPXV for example.

4) Immunization protocol transparency and consistency. The paper alternates between non-lethal and lethal VACV challenge models without clearly explaining the rationale. This lack of consistency makes interpretation harder. Clarifying why different challenge intensities were used and whether results are directly comparable would improve the narrative. Moreover, it is unclear whether: Protein doses were matched across all tested EFC components; Adjuvant concentration and formulation were standardized; Dosing schedules and number of boosts were consistent. The authors should clarify whether immunogenicity comparisons are valid across proteins.

Minor
Terminology: The manuscript repeatedly refers to “monkeypox” rather than “mpox,” which is now the WHO-recommended term. This should be corrected throughout to reflect current naming conventions.
Text redundancy and narrative flow: Flow of information can be difficult to follow; some critical experimental details or conceptual explanations are introduced only later in the manuscript.

Reviewer #3 (Remarks to the Author):

The authors present a manuscript entitled “Antibody Binding and Neutralizing Targets within the Predicted Structure of the Poxvirus Multiprotein Entry-Fusion Complex’ in which they identify neutralising antibody targets in the poxvirus entry complex and go on to use these targets to show that they might be good candidates for poxvirus vaccines.
Overall there are important observations in this paper, including the identification of A16 and G9 as targets of neutralising antibodies that can be induced by vaccination but not in the context of infection with whole virus. The authors also present a model of most of the entry complex using alpha fold 3 that sheds light on some of the entry complex function and how different types of antibody might interact with it. This manuscript has current relevance due to the requirement for better mpox vaccines. The manuscript is well written but there are however several points to be addressed before it is ready for publication. These are outlined below:

Figure 1: There should be measurements of protein purity, especially as proteins have been purified from serum-containing medium. Differences in purity will leads to differences in absolute mass of antigen injected into rabbits which can significantly influence the amount and quality of antibody generated, making comparisons between the sera generated against antigens hard to interpret.

Figure 1 C/D/E - are these data from single rabbits for each antigen? If so one recommendation is to use at least biological dupicates to be certain of the relative differences between antigens (especially considering the above comment about antigen purity).

Figure 2: The IPs (E and F) need a negative control protein eg. FLAG-GFP or a tagged non-binding VACV protein

Figure 3: It should be stated whether MVA expresses all the entry-fusion complex and whether there are major sequence differences in these proteins between VACV and MVA. Why are data from 3c using pooled sera and 3d using sera from individual mice? Figure 3F should include a positive control using a reagent that does inhibit VACV binding to cell surfaces.

Figure 5; The challenge experiments are convincing, but it would be helpful to link this protection to antibody function. Either the paper should acknowledge that protection can also be CD8 T-cell mediated and that the different vaccine antigens might elicit different CD8 memory responses (which is consistent with the data in 5C), or adoptive transfer experiments can be carried out to prove that the differences in protection levels between vaccine antigens, are at least in part, antibody-dependent (ie transferring serum from vaccinated mice into naive mice and challenging with VACV). Figure 5C the images are too small, suggest to move make them larger and move to supplementary data.

Figure 6: 6a, explain why pooled serum and how many were pooled in each case. The data presentation in 6a here is also confusing.

Figure 7: The model by Alphafold3 is interesting but its accuracy should be caveated in the text due to the lack of experimental validation. There are lots of protein/protein interaction predictions that could be tested with mutations to improve the confidence in what is otherwise a prediction with plenty of assumptions including the stoichiometry, the relative orientations, the degree flexibility of the individual chains etc… So either test the predictions or state that the assumptions that limit the confidence in accuracy.

****

Version 1:

Reviewer comments:

Reviewer #1

(Remarks to the Author)
The authors put significant effort into revising the manuscript, which has substantially improved the data. In particular, the discovery that the block to A16/G9 antibody induction by live virus can be overcome by deleting the A56/K2 complex was very nicely incorporated into the manuscript.
I also note that I intended to refer to M1 in my question: “Why is the combination with EV proteins not significantly better than the H3/EV combination when the single H3 versus the A16/G9 combination is considerably better?”
The authors have addressed all of my points, and I fully support the publication.

Reviewer #2

(Remarks to the Author)
The authors have substantially improved the manuscript in response to the reviewers’ comments. The revised version is much clearer, and the key points raised during the review process have been thoroughly addressed. I have no remaining major concerns.

Reviewer #3

(Remarks to the Author)
The authors have presented a revised version of their manuscript in which they have successfully addressed all of the questions points raised the original review and is suitable for publication.

Decision Letter:

26th February 2026

Dear Bernie,

Thank you for your patience while your manuscript "Identifying Neutralization Targets in the Poxvirus Entry-Fusion Complex Reveals A16/G9 as a Potent Vaccine Antigen Concealed by Viral Immune Evasion" was under peer review at Nature Microbiology. It has now been seen by our referees, whose expertise and comments you will find at the end of this email. Despite their enthusiasm and support for your manuscript, we have decided that we cannot offer to publish your manuscript in Nature Microbiology.

In particular, given that a recently published study overlaps strongly with your findings (doi: 10.1016/j.cell.2025.07.040) and although we acknowledge that some aspects of your study have not been described before, unfortunately, given the high volume of submissions we receive and the limited space in our pages, competition for publication in Nature Microbiology is extremely strong.
After editorial discussion, we feel that the novelty of the conclusions is reduced in light of the related Cell paper published last year, and the study no longer meets the threshold required for consideration at the journal.

Although we cannot offer to publish your manuscript, I have discussed your study and the reviewers' comments with our colleagues at Nature Communications. They have advised that they would, in principle, accept the work for publication in the journal and without further peer review. Should you wish to take up this option, please use the link to the Springer Nature manuscript transfer below.

Your handling editor at Nature Communications would be Dr. Madlen Luckner (Madlen.Luckner@nature.com). If there is anything you would like to discuss before transferring the paper and its reviews, please don't hesitate to contact her by e-mail.

Please note that Nature Communications is a fully open access journal. For information about article processing charges, open access funding, and advice and support from Springer Nature, please consult the Nature Communications Open Access page (www.nature.com/ncomms/open_access/index.html).

To transfer your manuscript please use our manuscript transfer portal. You will not have to re-supply manuscript metadata and files, unless you wish to make modifications. For more information, please see our manuscript transfer FAQ page.

I am sorry that we cannot be more positive on this occasion, but hope that you find the referees' comments helpful when preparing your paper for resubmission elsewhere.

Yours sincerely,

****

Reviewer Expertise:

Referee #1: Poxviruses, vaccines
Referee #2: Poxviruses, vaccines, humans
Referee #3: Poxviruses, vaccines

Reviewers Comments:

Reviewer #1 (Remarks to the Author):

The authors put significant effort into revising the manuscript, which has substantially improved the data. In particular, the discovery that the block to A16/G9 antibody induction by live virus can be overcome by deleting the A56/K2 complex was very nicely incorporated into the manuscript.
I also note that I intended to refer to M1 in my question: "Why is the combination with EV proteins not significantly better than the H3/EV combination when the single H3 versus the A16/G9 combination is considerably better?"
The authors have addressed all of my points, and I fully support the publication.

Reviewer #2 (Remarks to the Author):

The authors have substantially improved the manuscript in response to the reviewers' comments. The revised version is much clearer, and the key points raised during the review process have been thoroughly addressed. I have no remaining major concerns.

Reviewer #3 (Remarks to the Author):

The authors have presented a revised version of their manuscript in which they have successfully addressed all of the questions points raised the original review and is suitable for publication.

Version 2:

Decision Letter:

7th March 2026

Dear Bernie,

Thank you for your letter asking us to reconsider our decision on your Article entitled "Systematic Analysis of Neutralization Targets in the Poxvirus Entry-Fusion Complex Reveals A16/G9 as a Potent Antigen Masked by Viral Immune Evasion". Now that I have had a chance to discuss the matter carefully with my colleagues, I am sorry to have to tell you that we do not feel able to reverse our original decision.

Although we do appreciate the points you raise regarding how your findings complement those presented in Meola et al., our

view remains that the overlap in the main findings — specifically those we consider of broad interest to our readership — is too substantial for us to offer publication of your work in Nature Microbiology.

We would therefore encourage you to accept the offer from Nature Communications to not further delay publication of your study, as they have advised that they would, in principle, accept the work for publication in the journal and without further peer review. Should you wish to take up this option, please use the link to the Springer Nature manuscript transfer in my previous decision email. Should you encournter any issues with the link, please contact microbiology@springernature.com for assistance.

Your handling editor at Nature Communications would be Dr. Madlen Luckner (Madlen.Luckner@nature.com). If there is anything you would like to discuss before transferring the paper and its reviews, please don't hesitate to contact her by e-mail.

I am sorry that we cannot be more positive on this occasion. Unfortunately, the number of papers submitted to Nature Microbiology vastly exceeds the number that we can publish each month, and we are therefore frequently forced to make difficult decisions. I hope that you find our referees' comments helpful when preparing your paper for resubmission elsewhere.

Yours sincerely,

Version 3:

Decision Letter:

Our ref: NMICROBIOL-25051578C-Z

13th March 2026

Dear Bernie,

Thank you for submitting your revised manuscript "Systematic Analysis of Neutralization Targets in the Poxvirus Entry-Fusion Complex Reveals A16/G9 as a Potent Antigen Masked by Viral Immune Evasion" (NMICROBIOL-25051578C-Z).

Please first accept our sincere apologies for the confusion regarding your manuscript, as we have recently changed our policy regarding scooping protection. Nonetheless, we are upholding our promise of scooping protection as detailed in our previous decision letter and we want to thank you for highlighting this.

Therefore we'll be happy in principle to publish your work in Nature Microbiology, pending minor revisions to comply with our editorial and formatting guidelines.

We are now performing detailed checks on your paper and will send you a checklist detailing our editorial and formatting requirements in about a week. Please do not upload the final materials and make any revisions until you receive this additional information from us.

Thank you again for your interest in Nature Microbiology.

Please note that I will be on leave between 16th of March - 7th April and that another editor from the team will handle your manuscript in the meanwhile. Please do not hesitate to contact us if you have any questions and send them to microbiology@nature.com for now.

Sincerely,

Version 4:

Decision Letter:

13th May 2026

Dear Bernie,

I am pleased to accept your Article "Immunogenicity of poxvirus A16/G9 entry-fusion subcomplex and its restriction by A56/K2 protein informs vaccine design" for publication in Nature Microbiology. Thank you for having chosen to submit your work to us and many congratulations.

Over the next few weeks, your paper will be copyedited to ensure that it conforms to Nature Microbiology style. We look

particularly carefully at the titles of all papers to ensure that they are relatively brief and understandable.

Once your paper is typeset, you will receive an email with a link to choose the appropriate publishing options for your paper and our Author Services team will be in touch regarding any additional information that may be required. Once your paper has been scheduled for online publication, the Nature press office will be in touch to confirm the details.

You may wish to make your media relations office aware of your accepted publication, in case they consider it appropriate to organize some internal or external publicity. Once your paper has been scheduled you will receive an email confirming the publication details. This is normally 3-4 working days in advance of publication. If you need additional notice of the date and time of publication, please let the production team know when you receive the proof of your article to ensure there is sufficient time to coordinate. Further information on our embargo policies can be found here:
https://www.nature.com/authors/policies/embargo.html

After the grant of rights is completed, you will receive a link to your electronic proof via email with a request to make any corrections within 48 hours. If, when you receive your proof, you cannot meet this deadline, please inform us at rjsproduction@springernature.com immediately. You will not receive your proofs until the publishing agreement has been received through our system

Due to the importance of these deadlines, we ask you please us know now whether you will be difficult to contact over the next month. If this is the case, we ask you provide us with the contact information (email, phone and fax) of someone who will be able to check the proofs on your behalf, and who will be available to address any last-minute problems.

Acceptance of your manuscript is conditional on all authors' agreement with our publication policies (see https://www.nature.com/nmicrobiol/editorial-policies). In particular your manuscript must not be published elsewhere.

Authors may need to take specific actions to achieve compliance with funder and institutional open access mandates. If your research is supported by a funder that requires immediate open access (e.g. according to Plan S principles or the NIH public access policy) then you should select the gold OA route, and we will direct you to the compliant route where possible. Because authors warrant under our subscription licensing terms that they haven't committed to licensing any version of their article under a licence inconsistent with the terms of our agreement – including the applicable embargo period – publication under the subscription model isn't suitable for authors whose funders require no embargo.

If you have any questions about our publishing options, costs, Open Access requirements, or our legal forms, please contact ASJournals@springernature.com

An online order form for reprints of your paper is available at https://www.nature.com/reprints/author-reprints.html. All co-authors, authors' institutions and authors' funding agencies can order reprints using the form appropriate to their geographical region.

We welcome the submission of potential cover material (including a short caption of around 40 words) related to your manuscript; suggestions should be sent to Nature Microbiology as electronic files (the image should be 300 dpi at 210 x 297 mm in either TIFF or JPEG format). Please note that such pictures should be selected more for their aesthetic appeal than for their scientific content, and that colour images work better than black and white or grayscale images. Please do not try to design a cover with the Nature Microbiology logo etc., and please do not submit composites of images related to your work. I am sure you will understand that we cannot make any promise as to whether any of your suggestions might be selected for the cover of the journal.

You can now use a single sign-on for all your accounts, view the status of all your manuscript submissions and reviews, access usage statistics for your published articles and download a record of your refereeing activity for the Nature journals.

To assist our authors in disseminating their research to the broader community, our SharedIt initiative provides you with a unique shareable link that will allow anyone (with or without a subscription) to read the published article. Recipients of the link with a subscription will also be able to download and print the PDF.

As soon as your article is published, you will receive an automated email with your shareable link.

Congrats to you and your co-authors! I am looking forward to seeing it published.

With kind regards,

P.S. Click on the following link if you would like to recommend Nature Microbiology to your librarian
http://www.nature.com/subscriptions/recommend.html#forms

** Visit the Springer Nature Editorial and Publishing website at www.springernature.com/editorial-and-publishing-jobs for more information about our career opportunities. If you have any questions please click here.**

Open Access This Peer Review File is licensed under a Creative Commons Attribution 4.0 International License, which permits use, sharing, adaptation, distribution and reproduction in any medium or format, as long as you give appropriate credit to the original author(s) and the source, provide a link to the Creative Commons license, and indicate if changes were made.
In cases where reviewers are anonymous, credit should be given to 'Anonymous Referee' and the source.
The images or other third party material in this Peer Review File are included in the article's Creative Commons license, unless indicated otherwise in a credit line to the material. If material is not included in the article's Creative Commons license and your intended use is not permitted by statutory regulation or exceeds the permitted use, you will need to obtain permission directly from the copyright holder.
To view a copy of this license, visit https://creativecommons.org/licenses/by/4.0/

An overview of the revision, descriptions of added experiments, clarifications, and detailed point-by-point responses are shown below.

Overview

We greatly appreciate the opportunity provided by the editor to revise our manuscript and the reviewers' assessment that our study is significant and provides new insights into poxvirus biology and vaccine development, as well as their thorough evaluation and constructive feedback. Notably, an insightful comment from Reviewer #1 prompted a critical experiment that revealed a new role for the viral A56/K2 protein in suppressing the antibody response to the A16/G9 entry-fusion proteins. Several new experiments described below strengthened the major conclusions of the study, which are emphasized in the revised title: Investigating Neutralization Targets within the Poxvirus Multiprotein Entry-Fusion Complex Reveals A16/G9 as a Potent Vaccine Antigen Concealed by Viral Immune Evasion.

The major additions to the manuscript include the following:

- Immunofluorescence evidence demonstrating the binding of anti-A16/G9 antibodies to virions on the surface of infected cells (Fig. 2c)
- In vivo evidence that immunization with A16/G9 provides cross-protection against cowpox virus (CPXV) (Fig. 4d, f)
- Analysis showing vaccinated humans and macaques did not produce detectable anti-A16/G9 antibodies and only 1 of 5 macaques infected with monkeypox virus (MPXV) induced such antibodies (Fig. 5b, c, d)
- Evidence that MVA did not significantly boost antibodies primed by immunization with A16/G9 (Fig. 5e)
- Discovery that the block to antibody induction by live virus can be overcome by deleting the genes encoding the viral fusion suppressor A56/K2 complex, thereby revealing a previously unrecognized mechanism of viral immune evasion (Fig. 6f, g, h)

Other additions to address requests of the reviewers include:

- Binding and neutralization data from a second immunized rabbit (Fig. 1c, d, e)
- Inclusion of a positive control for the experiment showing that anti-A16/G9 antibodies do not prevent virion binding to cells, supporting the conclusion that these antibodies specifically block entry (Fig. 2g)
- Added significance indicators for neutralization, weight loss and survival data in figures 2 and 4
- Reorganization of Fig. 5a to better display and facilitate interpretation by reviewer and readers
- SDS-PAGE analysis of the entry fusion complex (EFC) proteins produced in insect cells (Extended Data Fig. 1a)
- Added Table to show the high conservation of EFC proteins and A56/K2 fusion suppressor (Extended Data Table 1)
- Added animal number (n) to the relevant figure legends
- Expanded introduction to describe the unique biology of poxviruses, including the existence of two infectious forms - the intracellular mature virion (MV) and the

extracellular enveloped virion (EV), the requirement for antibodies to both forms for complete protection, and the requirement for all 11 entry-fusion proteins for infectivity
- Expanded discussion of immune mechanisms and statement of study limitations

Reviewer #1 (Remarks to the Author):

This manuscript by Yu and coworkers aimed to investigate the structure of the poxviral entry fusion complex (EFC) and its correlation with the activation of neutralizing and non-neutralizing antibodies against EFC proteins. Several EFC proteins have been identified as inducing neutralizing antibodies, while others do not activate neutralizing antibodies but rather bind to them. In doing so, they identified the G9/A16 EFC protein complexes as a potent activator of neutralizing antibodies that also cross-neutralize CPXV and MPXV. A16 and G9 proteins, positioned at the apex of a predicted AlphaFold3 EFC model, not only induce cross-neutralizing responses but also confer protection in murine models. Notably, these antigens appear to be immunologically silent during natural infection, suggesting a potential mechanism of immune evasion. Moreover, the effect of the G9/A16 heterodimer complex overcame the impact of the individual proteins G9 and A16, and also synergized with the effects of EV proteins A35 and B6. Together, these insights provide a refined structural and immunogenic map of the EFC, advancing the rational design of next-generation poxvirus vaccines and identifying another mechanism of poxviral-induced immune evasion, which may also contribute to the development of improved strategies against poxvirus.
In summary, the significance of the data presented in this manuscript is great. These data will be interest for poxvirus biology in general but also for translation. These fascinating data significantly enhance our understanding of poxvirus biology and the mechanism of the entry-fusion process from a fundamental research perspective. The manuscript substantially increases our understanding of the mechanism and structure of EFC. In addition, another mechanism of poxviral immune evasion has been identified, which may also contribute to understanding the pathogenesis of poxviruses. These data will then allow for the targeted development of antivirals and other preventive measures, particularly in the context of orthopoxvirus vaccine development, which is an urgent need at present due to the ongoing outbreaks of mpox in Africa. In doing so, this manuscript serves as an excellent example of how basic research knowledge can be translated into practical applications.

Response: We appreciate the reviewer's positive assessment that our study is of great significance and advances our understanding of poxvirus biology and translational implications for orthopoxvirus vaccine development.

However, several points need more clarification and addition:
1. Mechanism of how antibodies to A16/G9 mediate neutralization and protection

Response: Thank you for this important question, which prompted us to clarify the mechanistic aspects in light of known poxvirus entry dynamics. Our previous published studies showed that the viral A16 and G9 proteins are required for core entry of vaccinia virus (VACV) into the cytoplasm to initiate infection (Ojeda et al. 2006; Laliberte et al. 2011). In Fig. 2g-h, we show

that antibodies to A16, G9 or the A16/G9 heterodimer do not prevent binding of virus to cells but rather inhibit virus entry either before or after virus binding to cells. We now point out that neither the mechanism of poxvirus membrane fusion nor the fusion peptides have been identified, preventing further mechanistic studies at this time (lines 374-376). We also acknowledge in the ***Limitations of the study*** *(lines 443-445) that potential contributions of T-cells, complement and ADCC have not been explored in the present study.*

- What is the explanation for the improved outcome of weight loss for the A16/G9 protein complex compared to the single proteins?

Response: Although weight loss was less following virus challenge of mice immunized with A16/G9 compared to the individual proteins, we now show that the maximum weight change of individual mice indicates the difference did not reach significance (Fig. 4c). Nonetheless, although we showed in Extended Data Fig.3c-f that the heterodimer elicits antibodies to both A16 and G9, it is possible that potent heterodimer-specific antibodies are also made (lines 178-179). In the future, we would like to do cryo-EM studies to determine the binding sites of anti-A16, anti-G9 and anti-A16/G9 polyclonal and monoclonal antibodies, which will clarify whether heterodimer-specific epitopes contribute to protection.

Why is there no complete resolution of weight loss?

Response: Thank you for highlighting this; we have expanded the description of immune responses to poxviruses. In the revised Introduction and Results, we cite previous reports showing that incomplete protection against weight loss follows immunization with proteins to the mature virion (MV) proteins alone and that combined responses to MV and extracellular enveloped (EV) proteins are required for complete protection against weight loss (lines 40-42, 275-280). Therefore, incomplete protection against weight loss following A16/G9 immunization was expected. However, we demonstrated complete protection against weight loss in mice immunized with A16/G9 plus EV proteins in Fig. 4i, consistent with the established role of EV-directed antibodies in limiting viral dissemination.

Why can the A16/G9, G9, and A16 not inhibit the initial round of viral replication, as seen by the photon emission in the head and chest? Why is there no benefit seen in the A16/G9 as seen in the outcome of morbidity? Does this correlate with titers of VACV measured in the organs?

Response: We appreciate this query relating to route-specific immunity, which aligns with known mucosal vs. systemic responses in virology. A16/G9 was administered subcutaneously (SC), a route that induces no or low neutralizing IgA in mucosal secretions. Mucosal administration of the vaccine might be more effective and could be tested in follow up studies, as suggested (lines 380-382). However, we did show that SC vaccination led to rapid reduction of virus replication in the head and greatly reduced spread from the site of inoculation to the body (Extended data Fig.6) thus reducing morbidity.

Regarding the second question, we cite previous studies (Luker and Luker 2008) demonstrating that the intensity of bioluminescence correlates well with VACV titers in organs (lines 264-265). Bioluminescence measurements have the advantage of following day-to-day spread in individual animals and reducing the numbers of animals required.

How do the antibodies inhibit the viral spread?
Response: In the revised Introduction, we explain that there are two forms of infectious poxvirus

– the intracellular MV and the extracellular EV and that the EV is essentially an MV with an additional outer membrane that was acquired prior to exocytosis and shields the EFC and other MV membrane proteins from antibodies (lines 32-40). In the Discussion (lines 382-387) we further explain that antibodies to EV membrane proteins can directly prevent virus spread, while antibodies to MV proteins can neutralize virus when the EV membrane is disrupted exposing the fusion apparatus in the MV - either upon the association of EVs with target cells, or following complement-mediated lysis of the EV membrane.

- What, in contrast, is the protective mechanism of MVA? Are there any immunodominant proteins identified in MVA?

Response: In the revised Introduction (lines 40-48), we explain that MVA and other attenuated vaccines induce neutralizing antibodies to the MV as well as EV, but depletion of the immunodominant MV antibodies does not substantially reduce the overall neutralizing activity of antiserum. It has been suggested that VACV induces low levels of antibodies to numerous proteins that together mediate neutralization. In the Limitations of the study, we point out that the contributions of T cells, complement and ADCC were not directly explored, and these remain important areas for future investigation (lines 443-445).

- Why is the combination with EV proteins not significantly better than the H3/EV combination when the single H3 versus the A16/G9 combination is considerably better?

Response: We are puzzled by the reviewer's question about H3 since we did not evaluate protection with this protein in the present study. Other studies show that H3, which is an immunodominant MV binding protein rather than an entry protein, only provides limited protection. If the reviewer intended to refer to M1, we did compare A16/G9 with EFC protein M1 (the MPXV homolog of VACV L1) and found that A16/G9 is significantly more immunogenic than M1 alone. Moreover, immunization with the combination of A16/G9 and EV proteins provided better protection against weight loss following virus challenge than the combination of M1 with EV proteins; only the A16/G9+A35+B6 group maintained and increased weight throughout the experiment, consistently exceeding 100% of the initial weight (Fig. 4h, i).

What is the mechanism of EV protein-mediated protection?

Response: In the revised Discussion (lines 382-387) we refer to evidence that antibodies to EV proteins can bind directly to EVs and inhibit virus spread in vitro and cite Vanderplasschen et al (1997) who presented evidence that some EV antibodies agglutinate virus on the surface of infected cells and prevent their release. Also, we previously reported that, in the presence of complement, antibodies against the VACV homolog of the A35 EV protein can lyses the EV membrane, allowing anti-MV antibodies to neutralize the exposed virus (Lustig et al. 2004).

- Are the cross-neutralizing antibodies also protective?

Response: We have strengthened this point with new data. We now provide in vivo evidence that that immunization with VACV A16/G9 protects mice against infection with CPXV (Fig. 4d, f), supporting cross-protection within orthopoxviruses. The same antibodies also neutralize MPXV (Fig. 2e) but because of personnel problems at NIH we were unable to do MPXV protection experiments, which require ABSL-3 containment. Nevertheless, consistent with the observed

protection against CPXV, MPXV A16/G9 and CPXV A16/G9 share high amino-acid sequence identity with their VACV counterparts (Extended Data Table 1), supporting the expectation of broader cross-protection pending future in vivo validation.

- Can we correlate the activation of strong nAb to their function in the EFC? Can we relate this to the essential or non-essential roles of these proteins in EFC?

Response: No direct correlation, but we've deepened the discussion with structural insights. Each EFC protein, even those that don't elicit neutralizing antibodies, is required for entry into cells and is conserved in all poxviruses as stated in the Introduction (lines 54-59). The study of Laliberte et al. 2008 suggested that some of the EFC proteins (A16, G9, F9, H2, O3) prevent the initial hemifusion step. while others (A28, L1, L5) only inhibit the later step of pore formation. However, these functional roles do not correlate with susceptibility to neutralization. Instead, our EFC modeling studies suggest that the spatial location of the proteins within the EFC may determine which components are preferential targets of neutralizing antibodies.

- If A16/G9 are blocked by nAb, can other EFC proteins compensate and further mediate fusion? Or is the conformation and functionality of EFC disturbed when single components are blocked?

Response: As stated in the Revised Introduction (lines 54-59), each EFC protein is required for entry, and each is conserved in all poxviruses. Thus, blockade of A16/G9 cannot be compensated by other EFC proteins; we also previously proved that gene deletion of any EFC component abolishes infectivity, reflecting disturbed function of the EFC.

- Is there a dosage effect of the A16/G9 protein immunization, such that if we increase the dosage, can we then block all EFCs initiated with a specific dosage of challenge virus?

Response: Good point for optimization; noted as future work. We show in Fig. 2h that antibodies to either A16 or G9 can prevent virus entry nearly 100% in vitro. Comparison of different in vivo dosages of A16/G9, as well as different adjuvants, would be important for optimizing vaccine regimens in future work.

- Can we induce the activation of A16/G9 antibodies after infection by using a kind of heterologous prime-boost vaccination strategy, that is, infection followed by protein-based vaccination with A16/G9? In a way similar to what is observed with the influenza virus and antigenic sin? Can a sublethal challenge dosage activate A16/G9 antibodies after infection?

Response: Good idea! We carried out a new experiment to determine whether MVA could boost antibodies to A16/G9 following a prime with soluble A16/G9. As shown in Fig. 5e, there was a slight increase that was not statistically significant. Thus, in our hands, live virus infection neither primes nor boosts antibodies to A16/G9.

- Is the very complex EFC itself also an immune evasion strategy when poxviruses apply more than 11 proteins for entry and fusion? At the same time, other viruses, such as measles or Coronaviruses, only use one protein to mediate fusion. Is the EFC also working when one or

more of the proteins are inhibited? Is that the explanation for the partial replication that was detected after BLI? If it is blocked, is the complex structure an advantage or disadvantage?

Response: Clarified that complexity increases potential targets rather than providing a mechanism for evasion. As explained in the Revised Introduction (lines 54-59), all EFC proteins are required for cell entry, increasing the potential number of antibody targets. Therefore, the large number of EFC proteins itself is not an immune evasion strategy. In support of this, we showed in Fig. 1 that antibodies to six of the EFC proteins are neutralizing. Minimizing the immunogenicity of the EFC and shielding the proteins from recognition by antibodies represent immune evasion strategies

2. A16/G9 immune evasion mechanism and impact on the EFC mechanism
- Is the immune evasion mechanism meaning that we did not detect A16/G9 ab after infection, also seen for MPXV infection? Are human sera from MPXV-infected individuals also negative for A16/G9 antibodies? Is there a difference between lethal and non-lethal infection? In the different MPXV clades?

Response: Data supports broad conservation across orthopoxviruses. Revised Fig. 5, panel a shows that neither attenuated MVA nor pathogenic VACV strain WR, which can cause lethal infection of mice, induce antibodies to A16/G9 in mice and rabbits. Panel b shows that the pathogenic CPXV strain Brighton, which causes lethal infection in mice, does not induce antibodies to A16/G9 in mice. Panel c shows that sera from humans vaccinated with the licensed Jynneos mpox vaccine did not have detectable A16/G9 antibodies. Panel d shows that sera from macaques vaccinated with Jynneos also did not have detectable antibodies to A16/G9. Panel d also shows that only one of five MPXV-infected macaques made detectable antibodies to A16/G9. We can confidently conclude that attenuated and pathogenic orthopoxviruses induce minimal or undetectable antibody response against A16/G9, across different hosts and viral strains.

- What is the impact of the A56/K2 protein complex on the immune evasion mechanism of EFC? Is there an effect of syncytia formation, that is, is there activation of A16/G9-specific antibodies after syncytia formation? What is the outcome of infection with A56 KO Virus?

Response: This was a highly insightful question, which led to the demonstration in a new experiment added as Fig. 6f, that a vaccinia virus A56/K2 deletion mutant induces robust anti-A16/G9 antibodies. Our previous experiments (Wagenaar and Moss, 2007, 2008; Wagenaar et al. 2008) showed that deletion of the genes encoding A56/K2 prevents the physical interaction of A56/K2 with A16/G9 and results in the "fuse-back" of progeny virus on the surface of cells, leading to insertion of the EFC into the cell membrane and cell-cell fusion, as diagrammed in Fig. 6h. We propose that exposed A16/G9 in the plasma membrane can be recognized by B cells, whereas A16/G9 in viral membrane is shielded from B cells.

- How was the evolution of this immune evasion mechanism? Was this also present in VARV?

Response: We showed in the revised Fig. 5 that antibodies to A16/G9 are undetectable or only occasionally induced by infections with VACV, CPXV and MPXV, suggesting that this phenomenon is widespread among orthopoxviruses (OPXV). The conservation of A16/G9 and A56/K2 in OPXV including VARV is shown in Extended Table 1. However, WHO strictly limits

experiments with VARV, which can only be carried out at the CDC or in a secure institute in Russia.

3. Correlates of Protection/Determinants of Vaccine-Induced Protection and Strategies for Broad Poxvirus Immunity

- If the A16/G9 are highly neutralizing, cross-reactive, and protective, but A16/G9 ab are not activated after MVA/VACV infection, what mediates the protection of MVA or VACV hyperimmune sera?

Response: Polyvalent antibodies. Live VACV (including MVA) induces antibodies to other immunodominant proteins, some of which are neutralizing in vitro and provide partial protection against weight loss in animal models. However, as stated in the Introduction (lines 42-48), depletion of these antibodies with individual antigens has only a small effect on the total neutralizing activity of immune serum. This has led to the proposal that VACV-induced serum neutralization is mediated by low amounts of neutralizing antibodies to many proteins, rather than by a few highly dominant targets. Antibodies to two EV proteins, VACV A33 and B5 (corresponding to MPXV A35 and B6 homologs, respectively), prevent virus spread in vitro and provide partial protection in animal models, but their importance following live virus infection has not been well studied.

- What is the impact of the infection route for the activation of A16/G9 ab after infection with VACV or MVA?

Response: The revised Fig. 5a shows that antibodies to A16/G9 were not induced by immunization with MVA intramuscularly in mice or in rabbits that were immunized percutaneously and boosted intravenously. Fig. 5b shows that antibodies to A16/G9 were not induced by infection with CPXV percutaneously. Fig. 5c shows that humans vaccinated subcutaneously with Jynneos (MVA) did not induce antibodies to A16/G9. Fig. 5d shows that rhesus macaques vaccinated with Jynneos subcutaneously do not make antibodies to A16/G9. Panel d also shows that only one of 5 rhesus macaques challenged intravenously with MPXV made detectable antibodies to A16/G9. Thus, across multiple routes (intramuscular, percutaneous, subcutaneous, and intravenous challenge), A16/G9 remains largely non-immunogenic in the context of live virus exposure.

- Can other EFC-proteins also mediate cross-protection?

Response: The EFC proteins are each highly conserved in all orthopoxviruses. Cross protection has been shown for VACV L1 and its MPXV homolog M1. Our data now extend such cross-reactive potential to A16/G9.

- What is the impact of the entry mechanism used? Are there differences between endosome-mediated entry and fusion-mediated entry in the activation of nAb to EFC, and also in the activation of A16/G9 proteins?

Response: The EFC proteins are required for entry by either route, as both routes require membrane fusion. It is possible that an antibody might have better access to the EFC when the

EV membrane is disrupted at the plasma membrane than in the endosome, but this has not been systematically studied.

- What is the impact of MVA dosages? Is the lower nAb also seen after 108 PFU immunization?

Response: We chose to use 10^7 PFU of MVA as a positive control, as this regimen has been shown to provide complete protection against OPXV challenges, as stated (lines 147-148). Formal dose-response studies for both A16/G9 and MVA would be important follow-up experiments for vaccine optimization.

- What is the protective impact of a single vaccination versus a first boost versus a second boost? How is that correlated with MVA?

Response: In this initial report of the immunogenicity of A16 and G9, we only determined protection after boosting. More detailed studies including dose, adjuvant, route of delivery, enhancement by expression from mRNA or as nanoparticles are worthwhile follow-up studies for publication in a vaccine-focused journal

- What is the cross-neutralization of a single vaccination? In principle, this should be the same after the first vaccination?

Response: We did not test cross neutralization or protection after a single vaccination in this initial study. Such experiments are indeed important and will be pursued in future vaccine related work.

- How could this be used to develop new antivirals for efficient blocking? Is this really effective, considering that inhibition of viral entry requires a very rapid application? how is that translated to the vaccine development? And how is the longevity?

Response: The structure of the A16/G9 heterodimer has been determined previously, which could facilitate the development of small molecule inhibitors. We agree that high affinity inhibitors would be needed and might only be effective at early stages of infection, but we do not feel such discussion is relevant to the current study.

Determination of longevity of protection, dosage and route of administration are all great experiments for future vaccine-development studies.

- How can AlphaFold be used to predict other promising antigens for vaccine development further? How could that be applied to new emerging poxviruses?

Response: The latest version of AlphaFold is better at predicting binding of antibodies and small molecules to proteins and could be used to engineer mAbs for better binding and develop therapeutics. Because of the high conservation of the EFC and other essential proteins, VACV structures could be used as a template to model the structures of other poxviruses if the amino acid sequences are determined.

Minor comments:
- Why are there different challenge dosages used?

Response: Rationale clarified. We used a 10^5 PFU challenge dose with VACV for experiments in which mice were immunized with MV proteins only and 10^6 PFU when immunized with MV and EV proteins because the combination provides greater protection as stated (lines 275-280).

- Ln 168 antibodies

Response: Corrected.

Reviewer #2 (Remarks to the Author):

This manuscript presents a carefully executed study investigating the immunogenic and structural properties of the poxvirus entry-fusion complex (EFC), an 11-protein assembly required for viral entry. The authors use AlphaFold3 modeling and recombinant protein immunization to identify new neutralizing antibody targets, specifically A16 and G9. These targets are not immunogenic during live virus infection but induce protective immunity when delivered as recombinant proteins. The experimental breadth is impressive, and the work provides new biological insights and relevant translational applications for vaccine development. However, some areas require clarification, including the consistency of protocols, statistical reporting, the translational relevance of the findings beyond VACV, and the organization and clarity of the manuscript itself. Nonetheless, this is a strong and impactful manuscript that makes a valuable contribution to the field.
Strengths
• Important biological target: The EFC is central to viral entry and poorly characterized immunologically, making this work timely and valuable.
• Novelty: Identification of A16 and G9 as highly immunogenic and protective components, despite their invisibility in natural infection.
• Robust experimental design: Thoughtfully designed experiments, including immunization and challenge studies in mice, supported by bioluminescent imaging and neutralization assays.
• Strong integration of structural modeling: Use of AlphaFold3 to map immunogenic sites is innovative and well-executed.
• Clear vaccine relevance: Results provide rationale for improving orthopoxvirus vaccines beyond Jynneos, especially in the context of mpox.

Response: We appreciate the reviewer's highly positive assessment of the importance, novelty, robust experimental design, and vaccine implications.

Major concerns
1) Paradox of highly protective but poorly exposed antigens: One central conceptual gap that needs further discussion is the apparent paradox that A16 and G9 are highly protective vaccine targets, yet seem to be immunologically silent during natural infection. If these antibodies confer protection in vivo, this implies that the antigens must be exposed at some point, either on the

virion surface or infected cells, to enable immune control.
The manuscript shows that recombinant A16 and G9 proteins induce potent neutralizing antibodies capable of inhibiting VACV, MPXV, and CPXV infection in vitro. In vivo, these antibodies confer protection against lethal VACV challenge, as demonstrated by reduced weight loss and improved survival in immunized mice. Structurally, AlphaFold3 modeling places A16 and G9 at the apex of the EFC, supporting the notion that they may be accessible on the virion surface. ELISA data suggest that antibodies against A16/G9 can bind to intact virions, although the extent of this binding is lower compared to other known immunogenic proteins.

However, despite these encouraging findings, the manuscript does not directly demonstrate surface exposure of A16 and G9 during natural infection. There is no imaging or biochemical evidence confirming their accessibility on native virions or infected cells.

Response: We agree that the apparent paradox between strong vaccine-induced protection and poor immunogenicity during natural infection is central to the conceptual impact of our work. We have now substantially expanded the relevant experiments and discussion. In addition to assessing the binding of neutralizing anti-A16/G9 antibodies to purified virions (Fig. 2b), we directly evaluated surface exposure by adding a new immunofluorescence experiment (Fig. 2c) showing binding of anti-A16/G9 antibody to unpermeabilized virions on the surface of infected cells.

The relative abundance of A16/G9 compared to major immunodominant antigens (such as A27, H3, or L1) is also not assessed and indicated, leaving it unclear how frequently these proteins are encountered by the immune system.

Response: Addressed with expert consultation. There are no published quantitative data on the abundance of EFC proteins in virions. However, we consulted Paul Gershon – the leading investigator on using mass spectrometry to analyze vaccinia virus proteins in virus particles – and he has given us permission to cite his unpublished findings that the major attachment proteins are 10 - 20-fold more abundant than EFC proteins (P. D. Gershon, personal communication) see lines 403-404.

Moreover, the temporal dynamics of their expression or exposure during the viral life cycle are not explored, making it difficult to assess whether antibodies could engage their epitopes in a timely manner to mediate protection.

Response: The Revised Introduction explains that the EFC proteins insert directly into membranes of assembling virus particles in the cytoplasm and do not traffic through the ER for display on the cell surface upon synthesis as occurs with many other viruses (lines 52-54). Therefore, exposure of antibodies to EFC proteins occurs only on the virus particle after exit from the cell.

While entry inhibition assays suggest functional blockade, the underlying mechanism—whether through binding to transiently exposed epitopes, structural interference within the EFC, or indirect effects—remains undefined.

Response: For the entry-inhibition assay, the infectious virus particles were incubated with anti-serum for 30 min at room temperature. For flaviviruses, transiently exposed epitopes only become available after prolonged incubation at 37°C and not at room temperature (VanBlargen

et al 2016). Therefore, we do not think that the epitopes on VACV MVs are only transiently exposed. However, during a natural infection the EFC in the MV membrane is shielded by the EV membrane so that exposure to anti-A16/G9 may only occur when the EV membrane is disrupted either upon binding to cells or following complement-mediated lysis as discussed (lines 382-389).

Since the mechanism of fusion and the fusion peptides of the multiprotein EFC have not been identified, it is not possible to distinguish direct inhibition of fusion from effects on structure of EFC (lines 373-376).

The lack of confirmatory surface staining of intact virions and absence of analysis of Fc-mediated effector functions (such as ADCC or phagocytosis) further limits the interpretation.

Response: As indicated above, we added an experiment (Fig. 2c) showing binding of antibodies to virions on the surface of unpermeabilized infected cells. We agree that further studies are needed, and we state that in the Limitations of the study, but we feel these are subjects for future investigations.

These gaps raise an important conceptual question: if these proteins are largely inaccessible during infection, how do vaccine-induced antibodies confer such robust protection? Additional experimental evidence is necessary to reconcile this paradox and fully establish the physiological relevance of A16 and G9 as vaccine targets.

Response: This is now extensively discussed in detail (lines 413-427). A16 and G9 are not inaccessible to antibodies as shown by binding to purified MVs (Fig. 2b, d) and to EVs with disrupted outer membranes (Fig. 2c) – presumably they are inaccessible to B cells so that antibodies to A16 and G9 are not produced during live infections. The question then is why antibodies to A16/G9 are induced by the soluble proteins but not by infection with live virus. We showed that a recombinant VACV expressing secreted G9 can induce antibodies (Fig. 6e) and in a new experiment (Fig. 6f) that deletion of the viral A56/K2 fusion suppressor genes, which results in the insertion of the EFC into the plasma membrane (Fig. 6h), confers the ability of live virus to induce antibodies to A16/G9. Taken together, these data imply that antibodies but not B cells can recognize A16/G9 in the virus particle. In the revised Discussion we cite published data indicating that A16/G9 is tightly bound in the viral membrane and interacts with a variety of other immunodominant proteins. Although antibodies and B-cell receptors are both immunoglobulins, the latter are membrane-bound with a tail that spans the B cell membrane into the cytoplasm, while antibodies are soluble immunoglobulins that lack membrane anchoring and a transmembrane tail. Because of this difference, free antibodies may better interact with antigen in 3-dimensions, whereas membrane bound B cell receptors may have more limited access in only two dimensions (Garcia-Sanchez et al. 2023 and references therein). This difference can explain how free antibodies can access A16 and G9 within the virus-associated EFC, while immobilized B-cell receptors are more restricted and can only recognize soluble A16 and G9 for antibody production. The next phase of our work will involve isolation and characterization of A16- and G9-specific B and T cells to test these ideas.

2) Lack of consistent statistical reporting. While the text refers to p-values and statistical tests,

the figures do not consistently include statistical annotations. In some key panels, there is no indication of which differences are significant. The authors should: Include statistical annotations (e.g., asterisks, exact p-values) on all figures where statistical tests were performed. Explicitly state in figure legends the statistical test used, sample size (n), and what comparisons were made.

Response: We thank the reviewer for highlighting the importance of consistent statistical reporting. We added statistical analysis for neutralization, weight loss and survival in Fig. 2 and 4.

3) Cross-protection claims should be nuanced. Although the paper frequently refers to orthopoxviruses broadly, all protection experiments were performed using VACV. Cross-neutralization of MPXV and CPXV are shown, but without in vivo validation. Since the authors show that neutralization does not always correlate with protection, the conclusion that these proteins confer cross-protection should be phrased more cautiously unless in vivo challenge data is provided for MPXV for example.

Response: We agree that in vivo cross-protection data are critical and have accordingly added a new CPXV challenge experiment. In a new experiment (Fig. 4d, f) we show that mice immunized with A16/G9 are protected from challenge with a lethal dose of CPXV. This directly demonstrates in vivo cross-protection beyond VACV. Unfortunately, due to personnel reductions at NIH, we were unable to access the ABSL-3 facility to do MPXV challenge. Nevertheless, consistent with the observed protection against CPXV, MPXV A16/G9 and CPXV A16/G9 share high amino-acid sequence identity with their VACV counterparts (Extended Data Table 1), supporting the expectation of broader cross-protection pending future in vivo validation.

4) Immunization protocol transparency and consistency. The paper alternates between non-lethal and lethal VACV challenge models without clearly explaining the rationale. This lack of consistency makes interpretation harder. Clarifying why different challenge intensities were used and whether results are directly comparable would improve the narrative.

Response: We appreciate this request for greater clarity. We used a 10^5 PFU VACV challenge dose for experiments in which mice were immunized with MV proteins only and 10^6 PFU when immunized with MV and EV proteins because the combination regimen provides greater protection and therefore permits evaluation under a higher challenge dose. We added this explanation (lines 275-280).

Moreover, it is unclear whether: Protein doses were matched across all tested EFC components; Adjuvant concentration and formulation were standardized; Dosing schedules and number of boosts were consistent. The authors should clarify whether immunogenicity comparisons are valid across proteins.

Response: The revised Methods now indicate that the same protocol was used in all experiments and the dose and timing are also now indicated in each figure legend. These details are now also summarized in each relevant figure legend to facilitate comparison of immunogenicity across antigens.

Minor
Terminology: The manuscript repeatedly refers to "monkeypox" rather than "mpox," which is now the WHO-recommended term. This should be corrected throughout to reflect current naming conventions.

Response: Mpox is the WHO recommended term for the human disease – not for the virus. The ICTV name for the virus remains MPXV. We have consistently used the WHO name for the disease and the ICTV name for the virus. This is similar to the use of smallpox for the disease and variola for the virus.

Text redundancy and narrative flow: Flow of information can be difficult to follow; some critical experimental details or conceptual explanations are introduced only later in the manuscript.

Response: We have substantially edited the manuscript to improve narrative flow. Key conceptual elements-such as the biology of MV and EV forms, the essential nature of all 11 EFC components-are now introduced earlier in the Introduction and then revisited with additional mechanistic context in the Results and Discussion. We have removed redundant passages and reorganized certain paragraphs to improve coherence and readability, while preserving all substantive content.

Reviewer #3 (Remarks to the Author):

The authors present a manuscript entitled "Antibody Binding and Neutralizing Targets within the Predicted Structure of the Poxvirus Multiprotein Entry-Fusion Complex' in which they identify neutralising antibody targets in the poxvirus entry complex and go on to use these targets to show that they might be good candidates for poxvirus vaccines.
Overall there are important observations in this paper, including the identification of A16 and G9 as targets of neutralising antibodies that can be induced by vaccination but not in the context of infection with whole virus. The authors also present a model of most of the entry complex using alpha fold 3 that sheds light on some of the entry complex function and how different types of antibody might interact with it. This manuscript has current relevance due to the requirement for better mpox vaccines. The manuscript is well written but there are however several points to be addressed before it is ready for publication.

Response: We appreciate the reviewer's comments on importance and relevance of our work. We recognize the need to address the key questions raised and have done so to the best of our ability in the revised manuscript prior to publication, as recommended by the reviewer.

These are outlined below:

Figure 1: There should be measurements of protein purity, especially as proteins have been purified from serum-containing medium. Differences in purity will leads to differences in absolute mass of antigen injected into rabbits which can significantly influence the amount and quality of antibody generated, making comparisons between the sera generated against antigens hard to interpret.

Response: We agree that protein purity is important for interpreting immunogenicity data. We now state (lines 477-478) and in the legend to Extended Data Fig. 1 that serum-free medium was used for protein expression, and the proteins were purified by binding and elution to Ni-NTA beads. Silver-stained gels of the purified proteins were added as in Extended Data Fig. 1a.

Figure 1 C/D/E - are these data from single rabbits for each antigen? If so one recommendation is to use at least biological dupicates to be certain of the relative differences between antigens (especially considering the above comment about antigen purity).

Response: Two rabbits were used for immunization with each protein. The revised Fig. 1c-e now shows the data from both rabbits.

Figure 2: The IPs (E and F) need a negative control protein eg. FLAG-GFP or a tagged non-binding VACV protein

Response: We thank the reviewer for this suggestion. In our co-immunoprecipitation experiments, A16-V5 serves as a internal negative control for G9-Flag, and G9-Flag serves as a internal negative control for A16-V5.

Figure 3: It should be stated whether MVA expresses all the entry-fusion complex and whether there are major sequence differences in these proteins between VACV and MVA.

Response: We now clarify that the 11 proteins of the EFC are conserved in all poxviruses (including MVA) as stated in the Introduction (lines 54-57). The added Extended Data Table 1 provides the amino acid identities of the homologs of 10 EFC proteins in VACV WR, MVA, CPXV, MPXV and VARV, confirming high conservation.

Why are data from 3c using pooled sera and 3d using sera from individual mice?

Response: We determined NT50 values for individual mice in the original Fig. 3d because we challenged individual mice. To conserve the small amounts of mouse sera, we pooled for Fig. 3e. We now explicitly state in the figure legends where pooling was used and for what purpose.

Figure 3F should include a positive control using a reagent that does inhibit VACV binding to cell surfaces.

Response: We repeated the experiment using high titer anti-VACV IgG as a positive control, which reduced binding by 50% (new Fig. 2g). At least four VACV proteins contribute to binding to cell surface glycosaminoglycans and we could find no reports of antibodies that inhibit binding. Nevertheless, the inclusion of this polyclonal positive control confirms that the assay can detect inhibition of attachment.

Figure 5; The challenge experiments are convincing, but it would be helpful to link this protection to antibody function. Either the paper should acknowledge that protection can also be CD8 T-cell mediated and that the different vaccine antigens might elicit different CD8 memory responses (which is consistent with the data in 5C), or adoptive transfer experiments can be carried out to prove that the differences in protection levels between vaccine antigens, are at least

in part, antibody-dependent (ie transferring serum from vaccinated mice into naive mice and challenging with VACV).

Response: We agree that both humoral and cellular immunity likely contribute to protection. In the revised manuscript, we explicitly acknowledge in the "Limitations of the study" that we did not evaluate T-cell responses, nor did we perform adoptive transfer experiments to formally demonstrate antibody dependence. We also note that antigen-specific CD8⁺ T-cell responses and serum-transfer studies would be valuable additions in future vaccine-related work to dissect the relative contributions of antibodies and T cells to protection.

Figure 5C the images are too small, suggest to move make them larger and move to supplementary data.

Response: As suggested, we moved the figure to Extended Data Fig. 6a, where they are presented at a larger size for improved visibility.

Figure 6: 6a, explain why pooled serum and how many were pooled in each case. The data presentation in 6a here is also confusing.

Response: The revised figure legends now indicate the number of sera pooled in each group. Pooling was used to conserve ELISA reagents and limited serum sample volume.

Figure 7: The model by Alphafold3 is interesting but its accuracy should be caveated in the text due to the lack of experimental validation. There are lots of protein/protein interaction predictions that could be tested with mutations to improve the confidence in what is otherwise a prediction with plenty of assumptions including the stoichiometry, the relative orientations, the degree flexibility of the individual chains etc… So either test the predictions or state that the assumptions that limit the confidence in accuracy.

Response: We added a caveat regarding the AlphaFold model to the Limitations of the study indicating that the model was not experimentally tested by structural or mutational analysis. In support of the model, we also include new co-immunoprecipitation data demonstrating the predicted interaction between the A16-G9 complex and A28 (Extended Data Fig. 2f). This provides experimental support for a specific predicted interaction, rather than validation of the full model.
